# ﻿A world generic revision of Quediini (Coleoptera, Staphylinidae, Staphylininae), part 1. Early diverging Nearctic lineages

**DOI:** 10.3897/zookeys.1134.87853

**Published:** 2022-12-08

**Authors:** Adam J. Brunke

**Affiliations:** 1 Agriculture and Agri-Food Canada, Canadian National Collection of Insects, Arachnids and Nematodes, 960 Carling Avenue, Ottawa, Ontario, Canada Agriculture and Agri-Food Canada, Canadian National Collection of Insects, Arachnids and Nematodes Ottawa Canada

**Keywords:** Integrative taxonomy, North America, rove beetles, Staphylininae

## Abstract

Several phylogenetically isolated, early diverging lineages of rove beetle tribe Quediini, all endemic to the western Nearctic, have recently been revealed by phylogenomic systematics. These three lineages, currently treated as either Quedius (Raphirus) or Q. (Paraquedius) warrant recognition at the genus level in the ongoing effort to achieve reciprocal monophyly of genera in Quediini. The three lineages were each morphologically studied in detail, with the following results: *Paraquedius* Casey, **stat. res.** is re-elevated to genus rank, *Quediellus* Casey, **stat. res.** is resurrected from synonymy and redefined, and *Iratiquedius***gen. nov.** is described for the species of the Amabilis and Prostans groups. A morphological diagnosis is provided for each genus at both the global and regional (Nearctic) level. Species level revisions, with keys, are provided for *Iratiquedius*, *Paraquedius*, and *Quediellus* with the following results: *Iratiquediusuncifer***sp. nov.** and *Paraquediusmarginicollis***sp. nov.** are described, *Quediellusnanulus* Casey is treated as **syn. nov.** of *Quediellusdebilis* (Horn), and *I.amabilis* (Smetana), *I.mutator* (Smetana), and *P.puncticeps* (Horn) are substantially redefined. Where possible, CO1 barcode sequence data are integrated with the morphological species concepts used herein and their clusters were found to be congruent.

## ﻿Introduction

The rove beetle tribe Quediini is a hyperdiverse, mainly northern hemisphere group of just more than 800 species that are predators of invertebrates in a variety of ecosystems including forests, alpine zones, and wetlands (Smetana 1971; Brunke et al. 2021). After a series of phylogenetic papers, which gradually reduced and redefined the lineage (reviewed in Brunke et al. 2021), a more restricted Quediini was recently extensively sampled for phylogenomic analyses and was demonstrated to be monophyletic, with the exception of the south temperate species that still formally remain in *Quedius* Stephens but instead belong to Amblyopinini pending generic revision (Jenkins Shaw et al. 2020). The final elements of *Quedius* shown to belong to other tribes by Brunke et al. (2021) were formally transferred out of Quediini and described as genera of Cyrtoquediini and Indoquediini by Brunke (2021).

**Figure 1.**
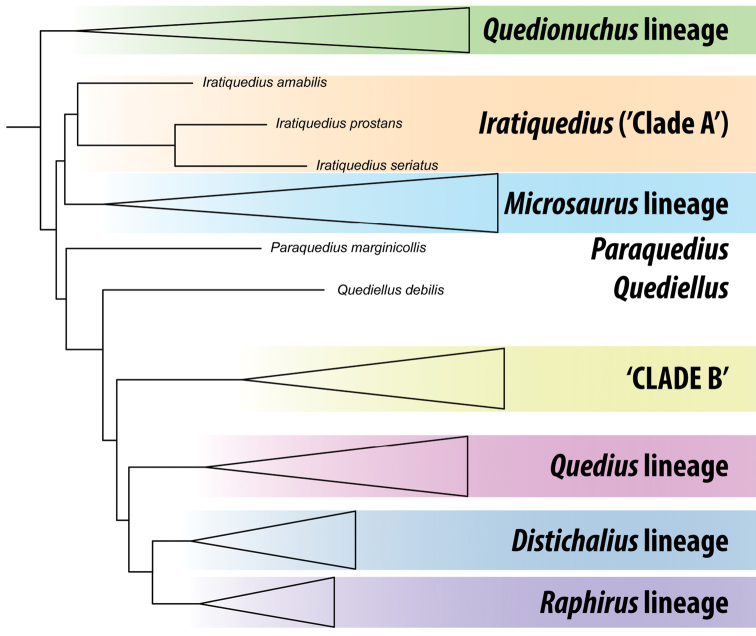
Schematic summary of Quediini (Staphylinidae: Staphylininae) topology recovered by Brunke et al. (2021) based on a partitioned maximum likelihood phylogenetic analysis of nearly 500 protein-encoding loci, and with taxonomy updated based on the results of the present paper.

Despite delimiting a morphologically well-defined and monophyletic Quediini, analyses by Brunke et al. (2021) also revealed that, unless all 800+ species of Quediini and its corresponding broad morphological diversity were treated as *Quedius*, an extensive generic revision will be required to achieve diagnosable, monophyletic genera. Aside from the *Quedionuchus* lineage, which consisted only of *Quedionuchus* Sharp and *Queskallion* Smetana, Quediini was resolved by Brunke et al. (2021) as four diverse clades (*Distichalius* lineage, *Microsaurus* lineage, *Quedius* lineage, and *Raphirus* lineage) and four smaller lineages (Fig. 1). Of these latter four, three of them are endemic to the Nearctic region and will be the topic of this first paper: ‘Clade A’ (here described as *Iratiquedius* gen. nov.), *Paraquedius* Casey, and the *Quediusdebilis* group (here treated as genus *Quediellus* Casey stat. res.). The final small lineage of Brunke et al. (2021), ‘Clade B’, includes the ‘Aenescens group’ of Smetana (1971), the ‘Daedalus group’ of Smetana (2017), and the species related to *Quediusriparius* Kellner. It is generally Holarctic in distribution and will be treated in a following contribution.

## ﻿Materials and methods

### ﻿Depositories

**BIO**Biodiversity Institute of Ontario, University of Guelph, Ontario, Canada (M. Pentinsaari);

**CNC**Canadian National Collection of Insects, Arachnids and Nematodes, Ottawa, Ontario, Canada;

**DEBU**University of Guelph Insect Collection, University of Guelph, Ontario, Canada (S. Paiero);

**FMNH**Integrative Research Center, The Field Museum of Natural History, Chicago, Illinois, USA (M. Thayer, M. Turcatel);

**MCZ**Museum of Comparative Zoology, Harvard University, Massachusetts, USA (C. Maier);

**UTCI**University of Tennessee at Chattanooga, Chattanooga, Tennessee, U.S.A. (S. Chatzimanolis).

### ﻿Specimen data

Type label data are given verbatim, with labels separated by “/” and comments indicated in square brackets. Non-type label data were standardized to improve clarity. Specimens were georeferenced using Google Earth or Google Maps.

### ﻿Microscopy, illustration, and photography

All specimens were examined dry using a Nikon SMZ25 stereomicroscope. Genitalia and terminal segments of the abdomen were dissected and placed in glycerin filled vials, pinned with their respective specimens. Line illustrations were made from standard images and then digitally inked in Adobe Illustrator CC-2021. All imaging, including photomontage was accomplished using a motorized Nikon SMZ25 microscope and NIS Elements BR v. 4.5. Photos were post-processed in Adobe Photoshop CC-2021.

Measurements were performed using the live measurement module in NIS Elements BR v4.5. Measurements were taken as listed below, but only proportional (HW/HL, PW/PL, EW/ EL, ESut/PL, PW/HW) and forebody measurements are stated directly in descriptions due to variability in body size. Total body length is generally difficult to measure accurately in Staphylinidae due to the contractile nature of the abdomen.

Abbreviations for measurements are as follows:

**HL** Head length, at middle, from the anterior margin of frons to the nuchal ridge;

**HW** Head width, the greatest width, including the eyes;

**PL** Pronotum length, at middle;

**PW** Pronotum width, greatest width;

**EL** Elytral length, greatest length taken from level of the anteriormost large, lateral macroseta to apex of elytra. Its length approximates the length of the elytra not covered by the pronotum and therefore contributing to the forebody length;

**EW** Elytral width, greatest width;

**ESut** Sutural length, length of elytral suture;

**Forebody**HL + PL + EL.

### ﻿Molecular data

Extraction, amplification, and sequencing of the barcoding fragment of CO1 were performed by the Canadian Centre for DNA Barcoding (CCDB), Biodiversity Institute of Ontario (BIO) (Guelph, Ontario, Canada). DNA was extracted from both dried museum specimens and 95% alcohol-preserved specimens. In some cases where Sanger-based methods failed to produce full-length sequences, re-attempts on existing DNA extracts were made with the NGS service of CCDB, which uses SMRT sequencing on a PacBio Sequel. Sequences were uploaded to BOLD and those sequences deemed to be barcode compliant by BOLD were assigned BINs (Barcode Index Numbers, Ratnasingham and Hebert 2013) and were considered as tentative species hypotheses. Using the Taxon-ID tree tool in the workbench of BOLD, barcodes and their associated BINs were visualized in a neighbour-joining tree using the BOLD aligner and Kimura-2 Parameter (K2P) distances. As the genera in question are phylogenetically isolated within Quediini and do not have clear sister groups among available barcode data, *Fluviphiruselevatus* (Hatch) (Indoquediini) was used to root the trees. The final dataset including voucher information is available on BOLD as the published dataset DS-QUEDREV1.

A sequence clustering analysis was performed on the dataset, using the workbench in BOLD to identify potential Operational Taxonomic Units (OTUs) using the refined single-linkage algorithm of Ratnasingham and Hebert (2013). This analysis is similar to that used to determine BINs but is useful to group sequences in advance of a BIN assignment or for those sequences that are too short to be BIN compliant. Results of the clustering analysis are reported with each species, under Comments. Phylogenetic analyses of the single-locus alignment using maximum likelihood were attempted but did not provide sufficient resolution to be useful, likely due to saturation at such deep divergences between the phylogenetically isolated lineages, including within genera. Monophyly of these clades was already demonstrated by phylogenomics (Fig. 1) (Brunke et al. 2021).

## ﻿Taxonomic account

### ﻿Staphylininae Latreille, 1802

#### ﻿ Quediini

Taxon classificationAnimaliaColeopteraStaphylinidae

Kraatz, 1857

A14594A8-F7BE-5558-9E4A-FAC941127A67

##### Diagnosis.

Quediini (as recently redefined by Brunke et al. (2021)) can be distinguished from other Staphylininae based on the following combination of characters: disc of head and pronotum with microsculpture, at least on lateral part of either head or pronotum; head with frontoclypeal punctures, and with posterior frontal and basal macropunctures (e.g., Fig. 3C) that are distinguishable from ground punctation by their larger diameter and longer, thicker setae; pronotum shield-shaped, slightly elongate to strongly transverse; profemora without apical row of lateroventral spines; protibiae without distinct subapical notch; all pretarsi with pair of empodial setae; all abdominal segments with only anterior transverse line (no traces of posterior transverse line), this line not encompassing spiracles (e.g., Fig. 3E, F).

#### ﻿ Iratiquedius gen. nov.

Taxon classificationAnimaliaColeopteraStaphylinidae

CE4046D5-DAF1-5D83-AC30-F5043F1172D7

https://zoobank.org/9DF5C736-97AD-48AD-9BDA-A49203711E06

**Figure 2.**
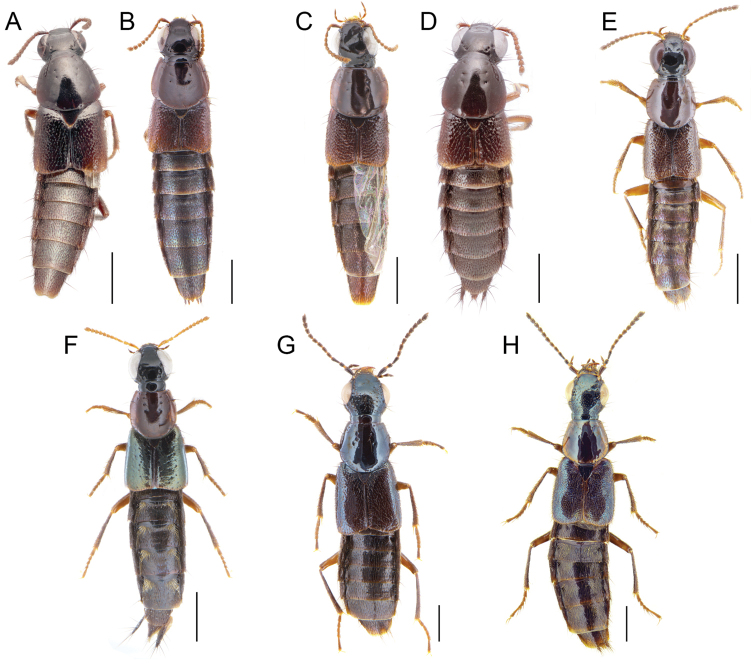
**A–H** dorsal habitus of **A, B***Iratiquediusamabilis* (Smetana) **A** male holotype **B** female non-type **C, D***I.mutator* (Smetana) **C** male non-type **D** female holotype **E***I.prostans* (Horn) **F***I.seriatus* (Horn) **G***Paraquediuspuncticeps* (Horn) **H***P.marginicollis* sp. nov. Scale bars: 1 mm.

**Figure 3.**
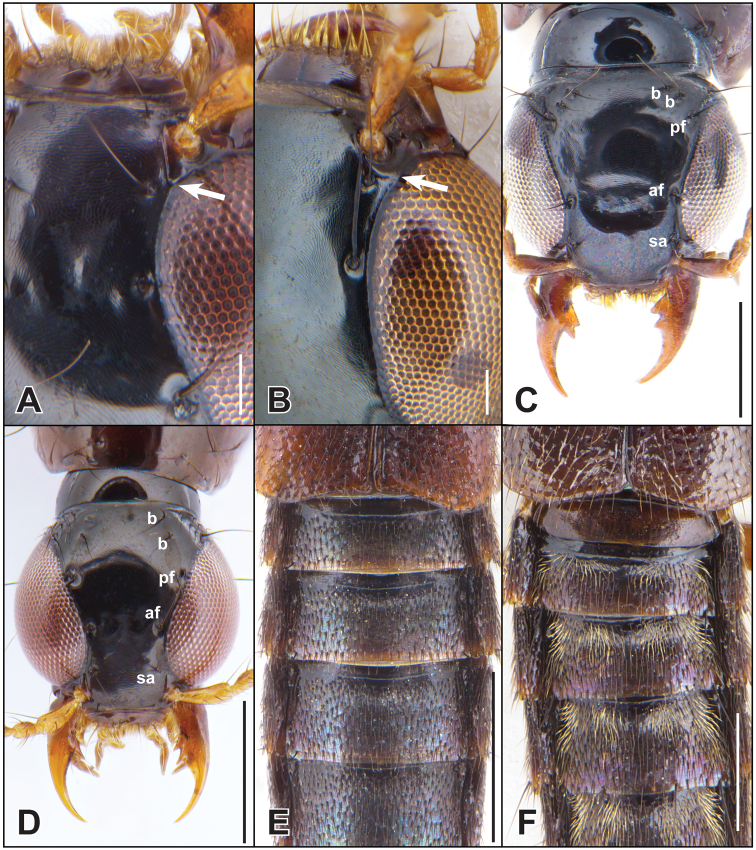
**A–D** dorsal head **A, B** showing confluence of inner eye margin and supra-antennal carina (arrow) **E, F** abdominal tergites **A, D***Iratiquediusseriatus* (Horn) **B**Quedius (Raphirus) probus (Casey) **C, E***I.amabilis* (Smetana) **F***I.prostans* (Horn). Abbreviations: af = anterior frontal puncture; b = basal puncture; pf = posterior frontal puncture; sa = supra-antennal puncture. Scale bars: 0.1 mm (**A, B**); 0.5 mm (**C–F**).

**Figure 4.**
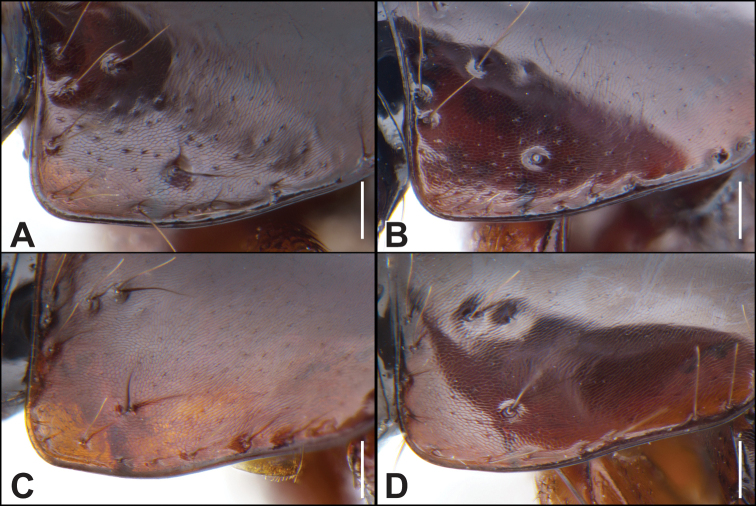
**A–D** anterior angle of the pronotum **A, B***Iratiquediusamabilis* (Smetana) **C, D***I.mutator* (Smetana) **A, C** macropterous morphotype **B, D** brachypterous morphotype. Scale bars: 0.1 mm.

Figs 2A–F , 3A, C–F , 4A–D , 6A–P , 7A–N , 9A–E

##### Type species.

*Quediusamabilis* Smetana, 1971.

##### Etymology.

The generic name is a combination of the Latin adjective ‘iratus’ and *Quedius*. It refers to the characteristic shape of the eyes, which are strongly convergent anteriad and create a comical, angry appearance.

##### Diagnosis.

Within Quediini, *Iratiquedius* can be distinguished from all other genera of the tribe by the distinctive eyes, which occupy nearly the entire lateral head margin, and are so convergent anteriad that their inner margin forms an obtuse angle with the suprantennal ridge (Fig. 3A). The global diagnosis is the same as the regional Nearctic diagnosis.

##### Description.

Medium to small rove beetles, with variable coloration (Fig. 2A–F). With the character states of Quediini (see Brunke et al. (2021)) and the following: antennomere 3 longer than 2, without dense setation; outer antennomeres (8–10) about as long as wide or shorter; antennae inserted close to inner margin of eye, separated by about the width of the antennal sclerite or less; head with eyes large, strongly convex, bulging from and nearly occupying entire lateral head outline, convergent anteriad and with inner margin forming obtuse angle with suprantennal ridge (Fig. 3A); with basal puncture doubled (at least one side), interocular punctures present in some individuals of some species (*I.amabilis*, *I.mutator*) or absent, paraocular punctures absent, genal puncture absent (Fig. 3C, D); frons not well-developed anterolaterad of antennal insertions; labrum notched medially, creating two lobes; apical maxillary and labial palpi fusiform and glabrous; infraorbital ridge complete to mandibles; gular sutures converging towards neck and narrowly spaced posteriad; mandibles with dorsal lateral groove absent or rudimentary, right mandible with single proximal tooth, tooth simple (Fig. 3D) or bifid (Fig. 3C); pronotum transverse to elongate, non-explanate, with three punctures in dorsal row, sublateral row at most reaching large lateral puncture, not extended posteriad; with only single large lateral puncture (e.g., Fig. 4A–D); hypomeron strongly inflexed, not visible in lateral view; basisternum with pair of macrosetae (reduced in *I.seriatus* and *I.prostans*) and well-developed longitudinal carina; scutellum impunctate; elytron with subbasal ridge complete and forming scutellar collar, disc without microsculpture between punctures; row of humeral spines present and well-developed; elytral punctation evenly distributed or in serial rows (*I.seriatus*); foretibia with lateral spines (reduced in *I.seriatus*, absent in *I.prostans*) and apical spurs; metatarsomeres with disc setose; metatibia with at least three spines on outer face; abdominal tergite I with prototergal glands developed as moderately deep impressions, outer margin with row of setae; abdominal tergites not deeply impressed at base but some species with paired median impressions causing a ‘pinched’ appearance; abdominal sternite III with basal transverse line forming obtuse angle at middle, not produced posteriad; aedeagus with well developed paramere bearing peg setae; at least some species with discrete, paired internal sac sclerites that may be homologous with the ventral paired sclerites described by Brunke et al. (2016) (e.g., *I.seriatus*, *I.uncifer* (Fig. 7H–K)).

##### Distribution.

*Iratiquedius* is endemic to western North America.

##### Bionomics.

Species of *Iratiquedius* are most often found in wet moss, though *I.prostans* is more of a generalist and can be collected from a variety of wet debris along running water.

##### Comments.

The three included species of *Iratiquedius* (*I.amabilis*, *I.prostans*, *I.seriatus*) were resolved together in ‘Clade A’ using a phylogenomic dataset (Brunke et al. 2021). Although Clade A was recovered by the concatenated but not coalescent analyses, the morphological configuration of the eyes, unique in Quediini, provides strong further evidence for its monophyly.

### ﻿Key to the species of *Iratiquedius*

**Table:** 

1	Microsculpture broken or missing on at least parts of pronotum; elytra with macropunctures arranged in rows, disc metallic blue to green but appearing brownish in greasy or teneral specimens (Fig. 2F)	**2**
–	Entire surface of pronotum with distinct, uninterrupted microsculpture; elytra with sparse to dense, even punctation, not arranged in rows, disc without metallic reflection (Fig. 2A–E)	**3**
2	Pronotum entirely without microsculpture, at most with a few short lines touching punctures; median lobe of aedeagus weakly produced ventrad in lateral view (Fig. 7F, G); ventral paired sclerites of internal sac with sharp, hooked apex (Fig. 7J, K); disc of female tergite X with distinct oval depression (Fig. 9E)	***I.uncifer* sp. nov.**
–	Pronotum microsculpture variable, ranging from evenly covered with broken transverse waves to entirely without microsculpture; median lobe of aedeagus strongly curved ventrad in lateral view (Fig. 7D, E); ventral paired sclerites of internal sac with rounded apex (Fig. 7H, I); disc of female tergite X evenly convex (Fig. 9D)	***I.seriatus* (Horn)**
3	Basal abdominal tergites with paired median impressions, creating a ‘pinched’ appearance, tergites and sternites with distinct patches of pale pubescence at base (Fig. 3F)	***I.prostans* (Horn)**
–	Abdominal tergites evenly convex, tergites and sternites without patches of pale pubescence at base (Fig. 3E)	**4**
4	Anterior angles of pronotum with shallow but distinct micropunctation (Fig. 4A, B); apex of median lobe in lateral view relatively short and rounded (Fig. 6A, B), in ventral view, apex slightly to distinctly emarginate (Fig. 6E, F); paramere with marginal row of peg setae remaining dense throughout (Fig. 6H, I); apex of female tergite X broader, with dense marginal setae (Fig. 9A); Sierra Nevada of California	***I.amabilis* (Smetana)**
–	Anterior angles of pronotum with barely perceivable micropunctation (Fig. 4C, D); apex of median lobe in lateral view relatively elongate and sharp (Fig. 6C, D), in ventral view, apex entire and pointed (Fig. 6G); paramere with marginal row of peg setae becoming sparse distally (Fig. 6J, K); apex of female tergite X sharply projected to a point, with sparse marginal setae (Fig. 9B); Coast Range Mountains and Central Valley of California	***I.mutator* (Smetana)**

#### ﻿ Iratiquedius amabilis

Taxon classificationAnimaliaColeopteraStaphylinidae

(Smetana, 1971) comb. nov.

E35D008D-421E-5A10-861E-FB0A1EF660D5

Figs 2A, B , 3C, E , 4A, B , 6A, B, E, F, H, I , 9A , 11A (map)

Quedius (Raphirus) amabilis Smetana, 1971: 205.
Quedius (Raphirus) amabilis : Smetana 1990 (distributional records).
Quedius amabilis : Brunke et al. 2021 (member of ‘Clade A’, non-Raphirus).

##### Type locality.

Near Strawberry, El Dorado County, California, United States.

##### Type material.

***Holotype*** (male, CNC): N.r. Strawberry, Eldorado Co. Cal., 16–17.61 [handwritten label] / #67, 45 [illegible, ‘mi camp’?] [handwritten label] /R. Schuster Collector [printed label] / HOLOTYPE *Quediusamabilis* Smetana 1968, CNC No. 10876 [red printed label] / CNC935809 [identifier label].

The aedeagus of the male holotype was never fully dissected by A. Smetana and was glued to the point with the specimen. For the present study, the aedeagus was relaxed, photographed and placed in glycerin within a genitalia vial.

##### Non-type material.

**United States: California**: Sierra Co.: 14 mi E Sierra City, Yuba Pass, 6,700’ [2,042 m], 26.VI.1976, L. & N. Herman (1 male, CNC); same except 26–28.VI.1976 (2 females, CNC).

##### Diagnosis.

*Iratiquediusamabilis* may be recognized within the genus by a combination of the evenly punctate elytra, the lack of golden setae or impressions at the base of the abdominal tergites and the distinctly impressed micropunctures on the anterior angles of the pronotum. The species most closely resembles *I.mutator* and can be distinguished from it by the distinct micropunctures of the anterior angles of the pronotum, the shorter apex of the median lobe in lateral view in males or broader, more densely setose apex of female tergite X.

##### Redescription.

Measurements ♂ (*n* = 2): HW/HL 1.21–1.22; PW/PL 1.29–1.32; EW/EL 1.28–1.31; ESut/PL 0.83; PW/HW 1.16–1.33; forebody length 3.3–3.4 mm.

Measurements ♀ (*n* = 2): HW/HL 1.18–1.21; PW/PL 1.17–1.24; EW/EL 1.45–1.59; ESut/PL 0.60–0.69; PW/HW 1.22–1.27; forebody length 2.9–3.0 mm.

Dark brown with pronotum becoming slightly to distinctly paler toward margin, elytra variably paler at base, sides and apically, in some individuals only scutellar area darkened, pale areas varying from brownish red to yellowish red or brownish yellow; antennomeres 1–3 sometimes slightly darker than remaining segments, which are brownish yellow to dark brown; pro- and mesofemora paler, yellowish brown; abdominal tergites narrowly paler apically, sternites with broader pale area at apex.

With distinct macropterous and brachypterous morphotypes (Fig. 2A, B). Head distinctly transverse, temples extremely short, immediately converging to neck posteriad of eyes; disc of head with microsculpture intermediate between transverse waves and transverse meshes, meshes becoming tighter on frons; posterior frontal puncture located at posterior fourth of eye; interocular punctures present or absent; labrum short, strongly transverse, forming two lobes; area between anterior frontal punctures with broad, transverse and shallow impression, encompassing interocular punctures, if present; antennomeres 1–5 (macropterous) or 1–4 (brachypterous) clearly elongate, segments becoming shorter toward apex, 8–10 weakly to moderately transverse; pronotum strongly (macropterous) to moderately (brachypterous) transverse; disc with microsculpture of transverse waves, often changing direction, becoming isodiametric meshes on anterior angles, anterior angles with distinct, shallow micropunctures, strongly (macropterous) (Fig. 4A) to moderately (brachypterous) impressed (Fig. 4B); elytra moderately (macropterous), to strongly transverse (brachypterous), at suture moderately (macropterous) to markedly (brachypterous) shorter than pronotum at midline, disc without microsculpture, punctation sparse, most punctures separated by about one puncture width (brachypterous) to slightly denser, with several punctures touching each other laterally (macropterous); abdomen with dense microsculpture of transverse waves, punctures slightly (macropterous) to distinctly (brachypterous) denser on bases of segments.

**Male.** Sternite VIII with distinct, wide V-shaped emargination; tergite X triangular, with short, rounded apex; sternite IX overall narrow, with long asymmetrical basal part and narrow, minutely emarginate apex; median lobe in ventral view with short tooth, subparallel, with slight expansion subapically, before converging to narrow, truncate apex bearing slight to distinct emargination (Fig. 6E, F); median lobe in lateral view arcuate ventrad, with moderately long apical area, wide tooth and narrow, rounded apex (Fig. 6A, B); paramere subparallel, slightly expanded subapically, converging to moderately narrow, rounded apex, with or without small emargination, peg setae arranged in dense marginal row (Fig. 6H, I).

**Female.** Tergite X narrowly triangular, with apex slightly attenuate, with dense marginal setae (Fig. 9A).

##### Distribution.

**United States**: CA.

This species is known only from two rather close localities in the Sierra Nevada of California.

##### Bionomics.

Nothing specific is known about this species’ microhabitat preferences, though it probably lives in moss along the margins of springs and spring-fed creeks.

##### Comments.

Smetana (1971) described *Quediusamabilis* and *Q.mutator* as the only members of the Amabilis group of Quedius (Raphirus). The former was known from one male and one female, both macropterous and collected from the Sierra Nevada, while the latter was only known from a single, brachypterous female collected at a different locality in the northern Coast Range mountains to the west. In addition to elytral and wing size, Smetana (1971) cited differences in the punctation of the abdominal tergites, shape of the antennomeres, elytral punctation and pronotum shape. Nothing was reported for many years until Smetana (1990), reported two brachypterous female specimens as *Q.mutator* and four macropterous males (one studied here) as *Q.amabilis*, both collected on Yuba Pass, California (Sierra Nevada) on different dates. He considered the possibility that *Q.mutator* may simply be a brachypterous morph of *Q.amabilis*.

A macropterous male from Yuba Pass was dissected and its aedeagus closely resembles that of the holotype of *I.amabilis* (Fig. 6A, B). The apical emargination of the median lobe in ventral view is less pronounced in the holotype compared to the non-type, and the paramere is slightly emarginate in the holotype, but these differences are considered to be intraspecific variation. In lateral view, the two specimens are nearly identical. The specimens are also similar externally and I agree with Smetana (1990) that they are conspecific. The two brachypterous females differ from the macropterous male in all other characters (antennae, pronotum, elytra, punctation of abdominal tergites) previously used to differentiate *I.amabilis* and *I.mutator*. One male and one female from the Yuba Pass series were sequenced and their half-length (325 bp) barcodes were only 0.34% divergent (Fig. 10). This result suggests that the two morphotypes collected together on Yuba Pass are conspecific, correspond to *I.amabilis* and they are here treated as such. The existence of a macropterous female (the unstudied allotype), if it is indeed a female, indicates that females of *I.amabilis* are wing dimorphic.

However, the female holotype of *I.mutator* differs from the Yuba Pass females by the even shorter elytra, shorter antennomeres 4–10 and the distinctly different apex of tergite X (Fig. 2D). This suggests that *I.mutator* is a valid species and that macropterous and brachypterous morphotypes confusingly exist in both species. All characters used previously to differentiate the two species are here considered to be associated with wing-dimorphism. Two fully winged males from the Central Valley of California were recently found in the FMNH collection (see below) and differ both externally (micropunctation of pronotum) (Fig. 2C) and in male genitalia from the known males of *I.amabilis*. One specimen was sequenced and its partial barcode was found to be 11.3% different from the Yuba Pass specimens (Fig. 10). Instead, these two males more closely resemble the female holotype of *I.mutator* in external morphology, and the concept of *I.mutator* is expanded below.

#### ﻿ Iratiquedius mutator

Taxon classificationAnimaliaColeopteraStaphylinidae

(Smetana, 1971) comb. nov.

79373CA5-B519-5C8F-A0F3-D1F0BF6F8C21

Figs 2C, D , 4C, D , 6C, D, G, J, K , 9B , 11A (map)

Quedius (Raphirus) mutator Smetana, 1971: 206.
Quedius (Raphirus) mutator : Smetana 1990 (distributional records, misidentification of Q.amabilis, in part).

##### Type locality.

8 miles north of Post Pile Camp [possibly ‘Valentine Spring’, ~ 1660 m], Tehama County, California, United States.

##### Type material.

***Holotype*** (female, CNC): 8 mi N Post Pile Camp, Tehema [Tehama] Co., Cal, VIII-30-60 [handwritten label] / R.O. Schuster Collector [printed label] / HOLOTYPE Quedius mutator Smetana 1968, CNC No. 10877 [red printed label] / CNC [handwritten label] / CNC93512 [identifier].

The female holotype has the shortest, most sparsely punctate elytra of all known individuals attributed here to either *I.mutator* or *I.amabilis*. The shape of female tergite X is unique and differs from the other examined females (here attributed to *I.amabilis*) by the projected, sharp apex (Fig. 9B).

##### Non-type material.

**United States: California**: Butte Co.: 3 mi NE Loma [Loma Rica], 39.354, -121.411, 151 m, 3.V.1981, sifting litter along spring, D. Chandler (2 males, FMNH).

##### Diagnosis.

*Iratiquediusmutator* may be recognized within the genus by a combination of the evenly punctate elytra, the lack of golden setae or impressions at the base of the abdominal tergites and the indistinct micropunctures on the anterior angles of the pronotum. The species most closely resembles *I.amabilis* and can be distinguished from it by the indistinct micropunctures of the anterior angles of the pronotum, the longer apex of the median lobe in lateral view in males or sharp, pointed apex of female tergite X.

##### Redescription.

Measurements ♂ (*n* = 2): HW/HL 1.22–1.25; PW/PL 1.20–1.24; EW/EL 1.37–1.38; ESut/PL 0.68–0.71; PW/HW 1.21–1.23; forebody length 3.4–3.5 mm.

Measurements ♀ (*n* = 1): HW/HL 1.20; PW/PL 1.22; EW/EL 1.70; ESut/PL 0.58; PW/HW 1.16; forebody length 2.8 mm.

Extremely similar to *I.amabilis*, and differing only in the following: antennomeres 1–4 (macropterous) or 1–3 (brachypterous) clearly elongate; pronotum moderately (macropterous) to weakly (brachypterous) transverse; pronotum with micropunctures of anterior angles indistinct (Fig. 4C, D); elytra overall shorter, and at suture relatively shorter than pronotum when comparing morphotypes; male sternite VIII with emargination vaguely more rounded at middle but varying from moderately narrow to as wide as *I.amabilis*; male tergite X more slender with narrower apex; male sternite IX broader, more strongly convergent to apex; apex of median lobe in lateral view longer, more elongate and sharp (Fig. 6C, D), median lobe in ventral view with apex entire and pointed (Fig. 6G); paramere with marginal row becoming sparser distally (Fig. 6J, K); apex of female tergite X sharply projected to a point, with sparse marginal setae (Fig. 9B).

##### Distribution.

**United States**: CA.

This species is known only from two localities: one in the mountains of the northern Coast Range and one in the Central Valley region.

##### Bionomics.

Both known localities are at least near springs, within areas of relatively dry, open woodland. Specimens of strongly hydrophilous species *I.uncifer* and *I.prostans* were co-collected at the type locality. The two non-type males were collected in litter along the margins of a spring. The holotype was collected at around 1600 m, while the non-type males were collected around 150 m.

##### Comments.

The concept given here for *Iratiquediusmutator* is considered to be a step forward but may need to be modified in the unlikely case that the population in the Central Valley is a third, undescribed species. Males from near the type locality will be needed to determine this with certainty.

#### ﻿ Iratiquedius prostans

Taxon classificationAnimaliaColeopteraStaphylinidae

(Horn, 1878) comb. nov.

5D0E804C-B517-50B4-BEAC-ACDFA7EAD003

Figs 2E , 3F , 6L–P , 9C , 11B (map)

Quedius prostans Horn, 1878: 165.
Quedius rupimontis Casey, 1915: 418.
Quedius (Raphirus) prostans : Smetana 1971 (redescription); Brunke et al. 2016, 2019, 2021 (phylogeny, outside of Raphirus).

##### Type locality.

‘California’, United States.

##### Type material.

***Lectotype* (male, MCZ)**: The lectotype of this common, widespread species was not examined as its identity was not in doubt.

##### Non-type material.

**Canada: British Columbia**: Central Kootenay: 8 mi W Creston, 10.VI.1968, Campbell and Smetana (8, CNC); Columbia-Shuswap: Mount Revelstoke National Park, 600 m, 17.VIII.1971, J.M. Campbell (1, CNC); Fraser Valley: 7 mi W Hope, 3.VI.1968, Campbell and Smetana (1, CNC); Squamish-Lillooet: Garibaldi Provincial Park, Diamond Head Trail, 1128 m, 1.VIII.1975, J.M. Campbell & B.A. Campbell (3, CNC); Garibaldi Provincial Park, Mimulus Creek, 1645 m, 8.VIII.1975, J.M. & B.A. Campbell (1, CNC); Mount Garibaldi, 30.V.1968, Campbell and Smetana (22, CNC); Vancouver Island: Elk Lake trail, 48.534470, -123.398647, 27.IX.2020, A. Davies, sedge litter at base of cottonwood, edge of lake (1, CNC); Goldstream Park, 27.Vl1968, A. Smetana (1, CNC); Goldstream Park, 5 mi N Victoria, 27.V.1968, Campbell and Smetana (21, CNC); same except, 6.VI.1975, JM & BA Campbell (3, CNC); Gabriola, sifting moss along pond edge, 3.V.1994, Lot 3, BF & JL Carr (1, CNC); same except Lot4, under wood on wet muck near pond (1, CNC); Nitinat Lake, at Caycuse River, 21.VI.1989, sifting wash up on beach, Lot 5, BF & JL Carr (2, CNC); Duncan, Mount Tzouhalem, 19.X.2008, A. Davies (2, CNC); Lake Cowichan, South Shore Road, 2.3 km N of town, wet moss, 16.VI.1979, I. Smith (9, CNC); Lake Cowichan, spring run beside North Shore Road, 1.7 km N town, moss and litter, 7.VI.1979, I. Smith (5, CNC); 10 mi E [Port] Alberni, MacMillan Provincial Park, 26.V.1968, Campbell and Smetana (1, CNC); near Mount Finlayson Trail, Malahat, Goldstream Provincial Park, moss on rock, 11.VII.1979, I. M. Smith (4, CNC); Port Alberni, Mount Arrowsmith, nr. road to ski area, 11.6 km off Highway 4, 28.VI.1979, I.M. Smith, moss on rocks and sticks in stream (5, CNC); Hillcrest Rd., 16 km S Mesachie Lake, along Lens Creek, 12.VII.2010, A. Davies (1, CNC).

**United States: Arizona**: Apache Co.: Chuska Mountains, Wagonwheel Campground, sifting leaf litter, 2250 m, 12.VII.1976, J.M. Campbell (5, CNC); same except: sifting moss along waterfall (28, CNC); **California**: Butte Co., 3 mi NE Loma, 3.V.1981, sift litter along spring, DS Chandler (1, FMNH); Mountain House [Brush Creek], 7.V.1981, sift litter along spring, DS Chandler (1, FMNH); Calaveras Co.: Big Trees State Park, 38.2775, -120.310556, 25.V-26.VI.2010, FIT, A.R. Cline & S.L. Winterton (12, UTCI); El Dorado Co.: 5 mi SW Kyburz, 1219 m, 6.V.1968, Campbell & Smetana (23, CNC); same except: 7 mi E Kyburz (6, CNC); Glenn Co.: 5 mi NE Alder Spring, 20.IX.1979, sift oak litter along spring, DS Chandler (2, FMNH); Humbolt Co.: Garberville, Garberville-Harris Road, 5–6 miles east of Garberville, 13.VII.1965, under stones and pieces of sod, Lot 2, BF & JL Carr (1, CNC); Los Angeles Co.: Mount Wilson, 600 m, 26.V.1978, J.O. Martin (1, CNC); Marin Co.: Point Reyes National Seashore, 2 mi W Inverness, 22.III.1983, A. Smetana (3, CNC); Mendocino Co.: Mendocino, 24.VI.1954, Helfer (2, CNC); Placer Co.: 4 mi S Truckee, Truckee River, 5.V.1968, Campbell and Smetana (1, CNC); Lake Tahoe, Tahoe City, 1950 m, 7.VII.1986, A. Smetana (1, CNC); San Bernardino Co.: San Bernardino Mountains, 1 mi NE Angelus Oaks, Cold Creek at Highway 38, 1828 m, 12.III.1983, A. Smetana (33, CNC); San Bernardino Mountains, Highway 38, 3 mi SW Onyx Summit, 2346 m, 14.III.1983, A. Smetana (13, CNC); San Diego Co.: Laguna Mountains, Little Laguna Lake, 5.III.1983, A. Smetana (10, CNC); Mount Laguna, Carex clumps at stream, 25.IX.1981, JM Campbell (3, CNC); Mount Palomar, 1524 m, sifting leaf litter, 27.IX.1981, J.M. Campbell (2, CNC); Mount Palomar, Fry Trail Campground [Fry Creek], 8.III.1983, A. Smetana (1, CNC); San Francisco Co.: San Francisco, 30.V.1911, Van Dyke (4, CNC); Siskiyou Co.: Calahan, Lillypad Lake, old rotten logs, 8.VII.1991, Lot 1,BF & JL Carr (1, CNC); McBride Springs, Mount Shasta, 1447 m, 20.VI.1974, A&D Smetana (1, CNC); Trinity Co.: 10 mi N Junction City, 762 m, 10.VII.1979, J.M. & B.A. Campbell (4, CNC); 12 mi N Junction City, 1030 m, 13.VII.1979, J.M. & B.A. Campbell (4, CNC); Upper Canyon Creek Meadows, 1463 m, 13.VII.1979, J.M. & B.A. Campbell (2, CNC); 19 mi W Coffee Creek Station, Shasta National Forest, 1219 m, 14.VII.1979, J.M. & B.A. Campbell (1, CNC); 4 mi W Forest Glen, 9.VII.1979, J.M. & B.A. Campbell (2, CNC); Tulare Co.: Wishon Campground, 12 mi NE Springville, Meadow Creek, sifting washed up debris in fast flowing creek, 21.VI.1993, Lot 11 BF & JL Carr (4, CNC); 28 mi NNW Kernville, Thompson Camp Spring, 1676 m, 30.V.1981, L. Herman (1, CNC); Tuolumne Co.: Strawberry, 3.VIII.1960, DG Cavanaro (3, CNC); **Idaho**: Boise Co.: 10 mi NE Idaho City, 10 Mile Campground, sifting moss, 18.VII.1981, J.M. Campbell (23, CNC); Elmore Co.: Boise National Forest, Ice Springs, 1463 m, 21.VII.1981, J.M. Campbell (1, CNC); middle fork of Boise River and Dutch Creek, 1370 m, sifting moss, 19.VII.1981, J.M. Campbell (20, CNC); **Nevada**: Douglas Co.: Zephyr Cove, 1900 m, 9.VII.1986, A. Smetana (13, CNC); **New Mexico**: Sandoval Co.: Sandia Mountain, Cibola National Forest, Las Huertas Creek, wet-soaked moss encrusted with calcareous deposits, springy slope, 8.VII.1981, A. Smetana (11, CNC); **Oregon**: Benton Co.: Mary’s Peak, 1158 m, 27.VII.1979, J.M. & B.A. Campbell (1, CNC); Mary’s Peak, waterfalls, 1066 m, 5.V.1973, E.M. Benedict (1, CNC); Clackamas Co.: Camp Creek, 3.5 mi SE Rhododendron, 700–730 m, 27.VI.1974, A&D. Smetana (2, CNC); Timberline Lodge Road, Mt. Hood, 28.VI.1974, A&D Smetana (1, CNC); Coos Co.: Dune Park, 3 mi N and 2 mi W North Bend, sunny frost pockets, 15.I.1972, E. M. Benedict (1, CNC); Curry Co.: Agness Rd., [crossing at Wake Up Rilea Creek, under stones and in little pools of water along shady, cascading creek, 10.VIII.1978, B.F. & J.L. Carr] (4, CNC); Deschutes Co.: 12 mi SW Sisters, 1341 m, J.M. & B.A. Campbell, 23.VII.1979 (11, CNC); Douglas Co.: 27.3 miles NE Reedsport, at Smith River Falls, 29.VI.1978, L & N Herman (2, CNC); Grant Co.: Dixie Summit, Highway 26, 1615 m, sifting moss, 22.VII.1981, J.M. Campbell (71, CNC); Malheur National Forest, 2 km NW Highway 26, Forest Road 1218, 1670 m, sifting moss, 22.VII.1981, JM & BA Campbell (13, CNC); Malheur National Forest, 7 km NW Highway 26, 2040 m, sifting old pile of hay, 22.VII.1981, JM & BA Campbell (2, CNC); road 2610, below Dixie Butte, 2050 m, 2.VI.1989, A. Smetana (8, CNC); Strawberry Range, Strawberry Campground, 1780 m, 1.VI.1989, A. Smetana (1, CNC); Strawberry Range, road 650, Fawn Spring, 1480 m, 30.V.1989, A. Smetana (16, CNC); Jackson Co.: highway 140, Little Butte Creek, 23.VI.1974, A.&D. Smetana (17, CNC); Klamath County: 13 mi NE Bly, near Deming Creek, 1706 m, 21.VII.1979, J.M. Campbell & J. Schuh (3, CNC); 16 mi NE Bly, Deming Creek Road, 1828, 21.VII.1979, J.M. Campbell & J. Schuh (9, CNC); 6 mi S Fort Klamath, Crooked Creek, 25.VI.1974, A.&D. Smetana (2, CNC); 9 mi NE Bly, Deming Creek, 1500–1760 m, A.&D. Smetana (6, CNC); Bly Mountain, 24.VI.1974, A.&D. Smetana (1, CNC); Gearhart Mountain, 1980–2194 m, 24.VI.1974, A.&D. Smetana (1, CNC); Sevenmile Creek, 1280 m, 20.VII.1979, J. Schuh & J.M. Campbell (2, CNC); Woodriver Springs, Jackson F. Kimball State Park, 25.VI.1974, A.&D. Smetana (15, CNC); same except, 1295 m, 20.VII.1970, JM Campbell (11, CNC); Mare’s Egg Spring, 1280 m, 20.VII.1979, J. Schuh & JM Campbell (1, CNC); same except, 25.VI.1975, A.&D. Smetana (7, CNC); Tecumseh Spring, 1280 m, 20.VII.1979, J. Schuh & J.M. Campbell (2, CNC); Tillamook Co.: 1 mi S Hebo, 28.VII.1979, J.M. & B.A. Campbell (2, CNC); Umatilla Co.: Umatilla National Forest, North Side Sugarloaf Mountain via Daniel Spring, mixed conifer forest, in wet moss at edge of spring/seep, 12.V.2012, A. Newton & M. Thayer (2, FMNH); 12 km NE Tollgate, Blue Mountain Road 63, 1250 m, A. Smetana (12, CNC); Union Co.: Blue Mountains, 9 km NW Elgin, Philips [Gordon?] Creek Road, 950 m, 27.V.1989, A. Smetana (22, CNC); same except 900 m, 25.V.1989 (3, CNC); Blue Mountains, Road 62, Jarboe Creek, 1200 m, 29.V.1989, A. Smetana (1, CNC); **Utah**: Cache Co.: Logan Canyon, 2 km N Wood Camp, sifting moss, 1706 m, 14.VII.1981, J.M. Campbell (36, CNC); **Washington**: Clallam Co.: 5 mi W Forks, 14.V.1968, Campbell and Smetana (1, CNC); Okanogan Co.: 8 mi NNW Republic, Sweat Creek [picnic area], 1097 m, 20.VII.1978, L&N. Herman (1, CNC); Pierce Co.: Mount Rainier National Park, Tahoma Creek, 700 m, 12.VIII.1973, A, Z & D Smetana (14, CNC); same except, 730 m, 10.VIII.1973 (2, CNC); Spokane Co.: 2 mi E Nine Mile, 13.IX.1955, R.A. Ward (1, CNC); Mount Spokane State Park, 1 km NE park entrance, 1000 m, sifting moss, 1.VIII.1981, JM Campbell (19, CNC); Mount Spokane State Park, Bald Knob campground, sifting moss, 1524 m, 31.VII.1981, JM & BA Campbell (1, CNC).

##### Diagnosis.

*Iratiquediusprostans* can be distinguished by a combination of elytra with even punctation, not arranged in rows, and pale pubescence at the bases of the abdominal tergites and sternites.

##### Redescription.

Measurements ♂ (*n* = 5): HW/HL 1.10–1.14; PW/PL 1.03–1.13; EW/EL 1.23–1.33; ESut/PL 0.65–0.76; PW/HW 1.05–1.15; forebody length 2.5–2.9 mm.

Measurements ♀ (*n* = 5): HW/HL 1.11–1.16; PW/PL 1.01–1.10; EW/EL 1.23–1.31; ESut/PL 0.69–0.78; PW/HW 1.06–1.09; forebody length 2.8–3.3 mm.

Head dark brown, pronotum and often elytra paler, dark reddish brown to reddish brown, abdominal segments broadly paler apically; antennae dark brown, antennomeres 1–3 with pale base; palpi reddish brown with apical segment dark brown; legs yellowish brown, tibia and metacoxae dark brown, tarsi brownish.

Head slightly transverse, appearing orbicular, temples extremely short, following outline of eye to neck; disc of head with moderately sparse microsculpture of transverse waves, becoming vaguely meshed in places, often completely meshed on frons, where it is denser; posterior frontal puncture located at posterior third of eye; interocular punctures absent; labrum short, transverse, forming two lobes; area between anterior frontal punctures with Y-shaped impression; antennomeres 1–4 or 1–5 elongate, 6–10 subquadrate, nine or ten, sometimes weakly transverse, antennomeres generally becoming shorter toward apex of antennae; pronotum roughly shield-shaped, subquadrate to slightly transverse; disc with microsculpture similar to that of head but becoming meshed on anterior angles; elytra appearing moderately to distinctly transverse; disc without microsculpture, evenly, moderately densely punctate, punctures generally closer than one puncture diameter but only sometimes touching, setae pale yellowish, appearing dark in greasy or wet specimens; abdominal tergites III–V, sometimes weakly on VI, with paired median impressions creating a ‘pinched’ appearance (Fig. 3F); tergites with paired patches of golden setae, one medial and one occupying entire basolateral corner; sternites with basal areas of golden setae; tergites with microsculpture of very fine and dense transverse waves; tergites with punctation varying from moderately dense at base to very sparse at apex.

**Male.** Sternite VIII with distinct, moderately deep and rounded emargination; tergite X elongate triangular to triangular, with several long marginal setae; sternite IX distinctly dilated at midlength, with long asymmetrical basal part and moderately deep emargination; median lobe in ventral view with one small, short, median tooth, apex truncate (Fig. 6L, M); median lobe in lateral view strongly narrowing to small apical part, apex rounded and with small ventral tooth, apical part projecting ventrad (Fig. 6N); internal sac with paired sclerites including a pair of slender, curved rod-like sclerites and a pair of broader fang-shaped sclerites (Fig. 6O); paramere longer than median lobe, elongate spoon-shaped in apical half, basal half markedly broad, apex narrowly rounded, peg setae arranged in single, elongate median field (Fig. 6P).

**Female.** Female tergite and sternite VIII with apex truncate to vaguely emarginate. Tergite X roughly pentagonal, with basal margin deeply incised, disc with faint to distinct, narrow median sulcus, apical area with inverted U-shaped darkening, apex slightly projected (Fig. 9C).

##### Distribution.

**Canada**: BC. **United States**: AZ, CA, CO, ID, MT, NM, NV, OR, UT, WA.

*Iratiquediusprostans* is the most widespread species of the genus. It occurs along the entire western cordillera, including both sides of the continental divide, and as far south as New Mexico in the east and near the United States border with Mexico, in the west. The species is not yet known from mountainous southern Alberta but is expected there.

##### Bionomics.

Although this species, like other *Iratiquedius*, seems to prefer moss, it has also been collected in other types of wet litter and even in rotting hay. This broader tolerance of microhabitats has likely allowed for a much wider distribution, across the drier forested areas of the western cordilleras to reach the eastern side of the continental divide.

##### Comments.

Specimens from across the distribution range were dissected and no consistent differences were observed in the aedeagus. This species varies enormously in size and in proportion of the body, giving the impression of multiple species. All specimens sequenced for CO1 barcodes, including those from both sides of the continental divide, were found to belong to a single cluster with 1.50% maximum pairwise distance (Fig. 10).

#### ﻿ Iratiquedius seriatus

Taxon classificationAnimaliaColeopteraStaphylinidae

(Horn, 1878) comb. nov.

A2615E20-2B5B-5C88-9021-A0F48FAD0B85

Figs 2F , 3A, D , 7A, B, D, E, H, I, L, M , 9D , 11C (map)

Quedius seriatus Horn, 1878: 166.
Quedius (Raphirus) seriatus : Smetana 1971 (redescription); Brunke et al. 2016, 2019, 2021 (phylogeny, non-Raphirus).

##### Type locality.

Vancouver, British Columbia, Canada.

##### Type material.

***Holotype* (male, MCZ)**: Van. [Vancouver] [printed label] / [male symbol] / Q. seriatus H. [handwritten label] / Type 7272 [red label]. Examined virtually.

The holotype male, although not dissected, was collected in Vancouver, British Columbia, Canada and far from the known distribution of *I.uncifer*. Therefore, the identity of this specimen is not in doubt.

##### Non-type material.

**Canada: British Columbia**: Fraser Valley: 7 mi W Hope, 3.VI.1968, Campbell and Smetana (2, CNC); Greater Vancouver: Stanley Park, 28.V.1968, Campbell and Smetana (1, CNC); Vancouver Island: 10 mi E [Port] Alberni, MacMillan Provincial Park, 26.V.1968, Campbell and Smetana (1, CNC); Port Alberni, Mount Arrowsmith, nr. road to ski area, 11.6 km off Highway 4, wet moss on rocks, 20.VII.1979, I.M. Smith (20, CNC); same except: moss on rocks and sticks in stream, 28.VI.1979 (10, CNC); Squamish-Lillooet: Garibaldi Provincial Park, Diamond Head Trail, 1128 m, 1.VIII.1975, J.M. Campbell & B.A. Campbell (3, CNC); Garibaldi Provincial Park, Mimulus Creek, 1645 m, 8.VIII.1975, J.M. & B.A. Campbell (2, CNC).

**United States: California**: El Dorado Co.: 5 mi SW Kyburz, 1219 m, 6.V.1968, Campbell and Smetana (5, CNC); Sierra Co.: 10 mi W Goodyears Bar, Hwy 49, under sandy-muddy moss clumps along cliff by a waterfall, 29.VI.1991, BF & JL Carr (1, CNC); Marin Co.: Point Reyes, 4.VI.1910, A. Fenyes (1 male, CNC); Siskiyou Co.: 5.4 mi SE Seiad Valley, O’Neil Creek, 457 m, 5.VII.1976, L & N Herman (1 female, CNC); **Oregon**: Benton Co.: Mary’s Peak, 1158 m, 27.VII.1979, J.M. & B.A. Campbell (8, CNC); same except 9.V.1968 (5, CNC); Mary’s Peak, 1066 m, waterfalls, 5.V.1973, E.M. Benedict (3, CNC); Clackamas Co.: mile 1 Timberline Lodge Road, 1463 m, 29.VII.1979, J.M. & B.A. Campbell (8, CNC); same except 28.VI.1974, A. & D. Smetana (2, CNC); Mt. Hood National Forest, Still Creek, Tributary at Highway 173, 1280 m, conifer forest, moss at stream edge, 15.V.2012, A. Newton and M. Thayer (1, FMNH); Douglas Co.: Scottsburg Bridge on Umpqua River, Hwy 38, moss, 11.XII.1971, E.M. Benedict (3, CNC); Hood River Co.: Mount Hood National Forest, Switchback Falls, 1340 m, 30.VII.1979, J.M. & B.A. Campbell (1, CNC); Mount Hood National Forest, Umbrella Falls, 1828 m, 30.VII.1930, J.M. & B.A. Campbell (2, CNC); Mount Hood National Forest, near Barlow Pass, 1220 m, 29.VI.1974, treading wet moss intermixed with low vegetation, muddy edge of small forest marsh, A. & D. Smetana (23, CNC); Jackson Co.: Highway 140, Little Butte Creek, 23.VI.1974, A. & D. Smetana (1, CNC); Tillimook Co.: 1 mi S Hebo, 28.VII.1979, J.M. & B.A. Campbell (1, CNC); **Washington**: Clallam Co.: 10 mi S Sequim, 12.V.1968, Smetana and Campbell (3, CNC); 5 mi W Forks, 14.V.1968, Smetana and Campbell (3, CNC); 6.5 mi N Sappho, 16.VII.1978, 365 m, L&N. Herman (1, CNC); 7 mi S Port Angeles, Sphagnum moss at water’s edge, 640 m, 14.VII.1975, A. Newton & M. Thayer (2, CNC); same except: 11.VIII.1979, J.M. & B.A. Campbell (4, CNC); 5 mi N Elwah Ranger Station, sifting moss and leaf litter along small stream, 12.VIII.1979, J.M. & B.A. Campbell (1, CNC); Jefferson Co.: Hoh Rainforest Ranger Station, 13.V.1968, Campbell and Smetana (4, CNC); Pierce Co.: Mount Rainier National Park, end of West Line Road, 3.VIII.1973, 1127 m, J.M. & B.A. Campbell (11, CNC); Mount Rainier National Park, Larrupin Falls, 1097 m, 3.VIII.1979, J.M. & B.A. Campbell (2, CNC); Mount Rainier National Park, Nisqually River, 1219 m, 16.V.1968, Campbell and Smetana (4, CNC); Mount Rainier National Park, Tahoma Creek, 730 m, 10.VII.1973, beaver ponds on creek, treading moss and grassy vegetation, A. & D. Smetana (9, CNC); Mount Rainier National Park, Van Trump Park Trail, 1645 m, 4.VIII.1979, J.M. & B.A. Campbell (1, CNC); Skamania Co.: Mount St. Helens, Spirit Lake, Bear Creek, 975 m, 6.VII.1974, A. & D. Smetana (1, CNC); Whatcom Co.: Mount Baker National Forest, Bagley Creek nr. Silver Creek Campground, 10.VII.1974, 609 m, A. & D. Smetana (1, CNC).

##### Diagnosis.

*Iratiquediusseriatus* can be distinguished from all other *Iratiquedius* except *I.uncifer* by a combination of: pronotum missing microsculpture on at least parts of the pronotum; elytra with serial punctation. From *I.uncifer*, it can be distinguished either by the rounded apices of the ventral paired sclerites of the internal sac, or the evenly convex disc of female tergite X.

##### Redescription.

Measurements ♂ (*n* = 5): HW/HL 1.06–1.11; PW/PL 1.00–1.03; EW/EL 1.22–1.26; ESut/PL 0.76–0.81; PW/HW 1.03–1.06; forebody length 2.3–2.6 mm.

Measurements ♀ (*n* = 5): HW/HL 1.07–1.10; PW/PL 1.01–1.04; EW/EL 1.22–1.27; ESut/PL 0.82–0.86; PW/HW 1.00–1.06; forebody length 2.4–2.9 mm.

Head dark brown; pronotum dark reddish brown, with sides often paler, red to reddish orange, some individuals with pronotum entirely pale reddish orange; elytra with metallic blue to greenish blue reflection, base, sides and apices often non-metallic, pale red to reddish orange; antennae yellowish brown, segments generally becoming darker toward the apex, segments usually with apices darker, antennomeres 6–11 often entirely dark brown; maxillary and labial palpi usually dark brown, sometimes brownish yellow with apical segment darker; legs brownish yellow, all femora, tarsi and metacoxae dark brown; abdominal tergites dark brown, sternites with broadly pale apices.

Head slightly transverse, appearing orbicular, temples extremely short, following outline of eye to neck; disc of head with sparse transverse waves, becoming vague meshes in places, frons with similar microsculpture but twice as dense; posterior frontal puncture located at posterior third of eye; interocular punctures absent; labrum short, transverse, forming two lobes; right mandible with single, simple tooth (Fig. 3D); area between anterior frontal punctures with Y-shaped impression; antennomeres 1–4 or 1–5 distinctly elongate, 6 and 7 subquadrate, 8–10 subquadrate to transverse; pronotum roughly shield-shaped to subparallel-sided, about as long as wide to scarcely wider than long; disc with variable microsculpture that is always broken or missing on at least some parts, or disc entirely without microsculpture; microsculpture when present consisting of transverse waves, often becoming meshes on anterior angles; elytra moderately transverse, slightly to markedly dilated at apex, slightly longer at suture relative to pronotum in females; disc with only serial rows of macropunctures in sutural, two discal, and one lateral row, disc without microsculpture; epipleuron with fine, evenly distributed setae; abdominal tergites III–V with slight basal impression, tergites III–VII with discrete patches of sparse, golden setae in a larger basal pair and smaller pair posterolaterad of basal pair, patches often blending together, golden setae often appearing brownish if specimen is greasy or wet; punctation of tergites sparse, generally becoming less dense toward apex and middle of disc, tergite VI–VIII very sparsely punctate in apical half, punctures separated by many times their diameter; tergites with dense microsculpture of transverse waves; sternites without patches of golden setae.

**Male.** Sternite VIII with distinct, moderately deep and rounded emargination; tergite X triangular to elongate triangular, with apical row of setae on or slightly removed from the margin; sternite IX overall moderately narrow to strongly dilated at midlength, with short to moderately long asymmetrical basal part and wide, with moderately deep to shallow emargination; median lobe without teeth, in ventral view with expanded subapical part delineated by a pair of marginal ridges, narrowing to rounded apex that is obtuse to acute, with pair of inner ridges (Fig. 7A, B); median lobe in lateral view strongly projected ventrad, slender to broadly triangular, ventral face evenly arcuate or with distinct bulges, apex sharp and narrow (Fig. 7D, E); internal sac with paired ventral sclerites, shape most apparent when everted, in situ with only apices showing, apices narrow and rounded, narrow part variable in length and curvature, expanded to wide rounded basal part (Fig. 7H, I); paramere about as long or slightly longer than median lobe, stout, subparallel to slightly converging apicad, with slightly to markedly emarginate, rounded apex, peg setae arranged in pair of longitudinal, slightly curved fields (Fig. 7L, M).

**Female.** Tergite X elongate triangular, with apex nearly truncate and subrectangular to narrowly rounded, disc evenly convex, with row of marginal setae (Fig. 9D).

##### Distribution.

**Canada**: BC. **United States**: CA, OR, WA.

##### Bionomics.

This species is strongly associated with water-soaked moss, especially growing along waterfalls and small, fast flowing creeks. Other specimens were collected in moss and other debris along the margins of forested marshes. *Iratiquediusseriatus* is known from a wide range of elevations ranging from near sea level (Stanley Park, BC) to 1828 m.

##### Comments.

Within *I.seriatus*, there is some variation in the length and curvature of the narrow, rounded end of the ventral paired sclerites within the internal sac (Fig. 7H–I), which seems to be consistent within a collecting event. The species is also unusually variable in the microsculpture of the pronotum, and shapes of male tergite X, sternite IX and the median lobe. Sequencing of additional CO1 barcodes is underway to establish whether molecular clusters correspond with this variation. With the barcode data available thus far (including two full length), a single OTU was identified by the cluster analysis, with 2.29% within-cluster variation (Fig. 10). This OTU cluster also contained a half-length sequence of *I.uncifer* and a subcluster of entirely *I.seriatus*, with little divergence between the two. The paramere may be distinctly emarginate in some individuals (Fig. 7M) but this is variable among co-collected specimens and is considered to be intraspecific variation.

#### ﻿ Iratiquedius uncifer sp. nov.

Taxon classificationAnimaliaColeopteraStaphylinidae

DCEE72BC-E6D1-541B-AB67-C8117FBEECAF

https://zoobank.org/1777598B-4305-47ED-9485-B6F273A87720

Figs 7C, F, G, J, K, N , 9E , 11C (map)

Quedius (Raphirus) seriatus : Smetana 1971 (partial misidentification).

##### Type locality.

Bear Creek (~4.5 km north of Dedrick), ~19.3 km north of Junction City, Trinity County, California, United States.

##### Type material.

***Holotype* (male, CNC)**: 12 mi N Junction City, Bear Creek, 3110’, 13.VII.1979, J.M. & B.A. Campbell [printed label] / CNC1014441 [identifier] / HOLOTYPE *Iratiquediusuncifer* Brunke sp. nov., des. Brunke 2022 [red printed label] ***Paratypes* (69, CNC). United States: California**: Humboldt Co.: Hydesville, VI.1901, Van Dyke (1 female, CNC); Sonoma Co.: Duncans Mills, 18.VII.1908, F.E. Blaisdell (1 female, CNC); Tehama Co.: 8 mi N post pile camp, 30.VIII.1960, R.O. Schuster (2 females, CNC); Trinity Co.: 10 mi N Junction City [possibly near Ripstein Campground], 762 m, 10.VII.1972, J.M. & B.A. Campbell (11, CNC); 12 mi N Junction City, Bear Creek, 1036 m, 13.VII.1979, J.M. & B.A. Campbell (34, CNC); 16 mi N Junctional City, Upper Canyon Creek Meadows, 1463 m, 13.VII.1979, J.M. & B.A. Campbell (2, CNC); 4 mi W Forest Glen, 9.VII.1979, J.M. & B.A. Campbell (19, CNC).

All specimens with: PARATYPE *Iratiquediusuncifer* Brunke sp. nov., des. Brunke 2022 [yellow printed label].

##### Etymology.

The species epithet refers to the diagnostic hooked apex of the ventral paired sclerites of the internal sac.

##### Diagnosis.

*Iratiquediusuncifer* can be distinguished from all other *Iratiquedius* except *I.seriatus* by a combination of: pronotum entirely without microsculpture; elytra with serial punctation (Fig. 2F). From *I.seriatus*, it can be distinguished either by the sharp, hooked apices of the ventral paired sclerites of the internal sac, or the distinctly impressed disc of female tergite X.

##### Description.

Measurements ♂ (*n* = 5): HW/HL 1.07–1.11; PW/PL 1.01–1.08; EW/EL 1.17–1.32; ESut/PL 0.76–0.93; PW/HW 1.01–1.07; forebody length 2.2–2.4 mm.

Measurements ♀ (*n* = 5): HW/HL 1.06–1.10; PW/PL 1.03–1.08; EW/EL 1.19–1.29; ESut/PL 0.74–0.87; PW/HW 1.01–1.06; forebody length 2.2–2.6 mm.

Extremely similar to *I.seriatus* and overlapping in most characters except: specimens of *I.uncifer* on average smaller, more slender, most examined specimens with bright orange-red pronotum and greenish elytra; pronotum entirely without microsculpture, at most with a few fragments of lines around the punctures; median lobe in ventral view with apical ridge (Fig. 7C); median lobe in lateral view less projected ventrad (Fig. 7F, G); internal sac with ventral paired sclerites sinuate, broad at base, strongly converging toward narrow, hooked apex (Fig. 7J, K); paramere with emargination smaller or absent, peg setae fields more truncate at base (Fig. 7N); female tergite X with long oval impression on disc occupying about half of its length (Fig. 9E).

##### Distribution.

**United States**: CA.

This species is currently known only from a cluster of localities in the Klamath Mountains and Coast Ranges of California.

##### Bionomics.

Nothing specific is known about this species’ preferred microhabitat but based on the locality data, it probably lives close to the edge of streams and springs. The specimens from Trinity County were co-collected with *I.prostans* and *Paraquediusmarginicollis*, indicating a very wet microhabitat along the edge of running water.

##### Comments.

Based on the material at hand, *I.uncifer* has yet to be collected together with *I.seriatus*. More collecting is needed in California to determine whether these two species are sympatric and if so, whether they occupy different microhabitats. A few females from the material originally examined by Smetana (1971) were found to belong to *I.uncifer*. This species was likely overlooked by Smetana (1971) due to the large amount of external variation in *I.seriatus* and *I.uncifer*, in addition to the fact that morphology of female tergite X was not yet routinely examined. The single half-length barcode sequence of this species was scarcely divergent from all available sequences of *I.seriatus* (Fig. 10) and it is not clear whether these two species will be reliably diagnosed using CO1 barcodes given further sampling. However, they are easily distinguished using the morphological features provided under Diagnosis.

#### ﻿ Paraquedius

Taxon classificationAnimaliaColeopteraStaphylinidae

Casey, 1915 stat. res.

B2FA19DD-7046-543E-A676-EFE1D05A329F

**Figure 5.**
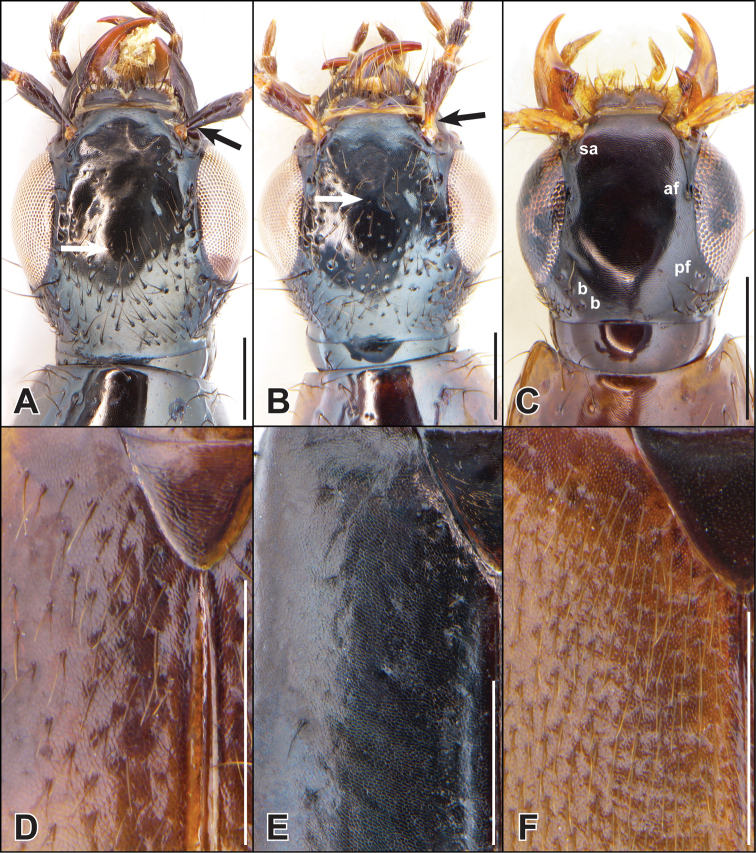
**A–C** dorsal head **A, B** showing extent of head punctation (white arrow) and color of first antennomere base (black arrow) **D–F** elytral punctation and microsculpture **A***Paraquediuspuncticeps* (Horn) **B***P.marginicollis* sp. nov. **C, D***Quediellusdebilis* (Horn) **E***Quedionuchuslongipennis* (Mannerheim) **F***Quediusdensiventris* (Casey). Abbreviations: af = anterior frontal puncture; b = basal puncture; pf = posterior frontal puncture; sa = supra-antennal puncture. Scale bars: 0.5 mm.

Figs 2G, H , 5A, B , 8A–J , 9F–G

Paraquedius Casey, 1915: 397, 400.
Quedius (Paraquedius) : Leng 1920; Hatch 1957 (characters); Smetana 1971 (redescription); Solodovnikov 2006 (phylogeny); Brunke 2016 (phylogeny); Brunke et al. 2019 (phylogeny), 2021 (phylogeny, non–Quedius, to be re-instated as genus).

##### Type species.

*Quediuspuncticeps* Horn, 1878.

##### Diagnosis.

*Paraquedius* is easily recognized within Quediini by a combination of the dark metallic blue/green reflections on the forebody, the punctate head and punctate scutellum. Within the Nearctic Region, *Paraquedius* is the only genus of Quediini with the disc of the head evenly punctate, at least on the posterior half. Worldwide, *Paraquedius* is superficially similar to the members of ‘Clade L’ of Brunke et al. (2021) (the Oriental and Palaearctic Multipunctatus and Intricatus groups of Quedius (Raphirus)), which are also metallic blue or green and have extensive head punctation. However, the latter have an impunctate scutellum, larger eyes and shorter appendages. *Paraquedius* is also superficially similar in habitus to members of the West Palaearctic clade (part of ‘Clade B’, Brunke et al. 2021) that consists of *Quediusriparius* and its close relatives. However, in *Paraquedius* the head is evenly punctate on the posterior half and the scutellum is punctate.

##### Redescription.

Medium sized rove beetles, with metallic blue to greenish forebody and long appendages (Fig. 2G, H). With the character states of Quediini (see Brunke et al. 2021) and the following: antennomere 3 longer than 2, with dense but not tomentose setae; all antennomeres longer than wide; head with eyes large, slightly more than twice as long as temples, convex, bulging from lateral head outline, subparallel and with inner margin well separated from suprantennal ridge (Fig. 5A, B); antennal insertions distant from inner margin of eye, separated by about 1.5 times the width of antennal sclerite (Fig. 5A, B); frons well developed anterolaterad of antennal insertions (Fig. 5A, B); head chaetotaxy obscured by the presence of many setae, though with clearly present anterior and posterior frontal punctures and frontal area glabrous, with interocular setae absent, genal puncture absent; labrum notched medially, creating two lobes; apical maxillary and labial palpi fusiform and glabrous, penultimate maxillary palpomere setose, some setae quite long, extending to about half length of apical maxillary palpomere; infraorbital ridge complete to mandibles; gular sutures converging towards neck and narrowly spaced posteriad; mandibles with dorsal lateral groove; right mandible with single bifid proximal tooth; pronotum subquadrate; hypomeron slightly inflexed, partly visible in lateral view; with only single large lateral puncture; dorsal row of pronotum with three to five punctures; sublateral row not reaching level of large lateral puncture; basisternum with pair of macrosetae though surrounded by many shorter setae, with longitudinal ridge on apical half; elytron with subbasal ridge complete, forming scutellar collar; scutellum punctate; row of humeral spines reduced to short row of darker, shorter regular setae; disc of elytra with even punctation, without microsculpture; foretibia with lateral spines and apical spurs; metatarsomeres with disc setose; metatibia spinose with at least three spines on outer face; abdominal tergite I with prototergal glands weakly developed as shallow impressions, impressions surrounded by some setae; abdominal tergites without impressed, glabrous basal areas; abdominal sternite III with basal transverse carina forming obtuse angle at middle, not produced; abdominal sternite VII unmodified; abdominal sternite VIII with distinct median emargination but emargination lacking membranous extension; aedeagus with well-developed paramere bearing peg setae (Fig. 8E, F, I, J).

**Figure 6.**
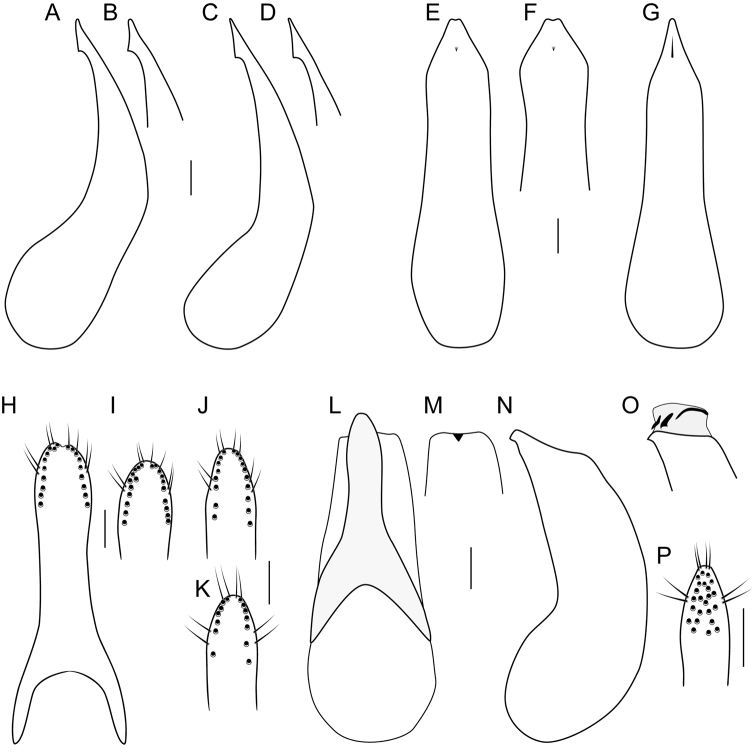
**A–G, L–O** median lobe of aedeagus **A–D, N, O** in lateral view **E–G, L, M** in ventral view **O** with internal sac everted **H–K, P** underside of paramere showing peg setae **A, B, E, F, H, I***Iratiquediusamabilis* (Smetana) **C, D, G, J, K***I.mutator* (Smetana) **L–P***I.prostans* (Horn). Scale bars: 0.1 mm.

**Figure 7.**
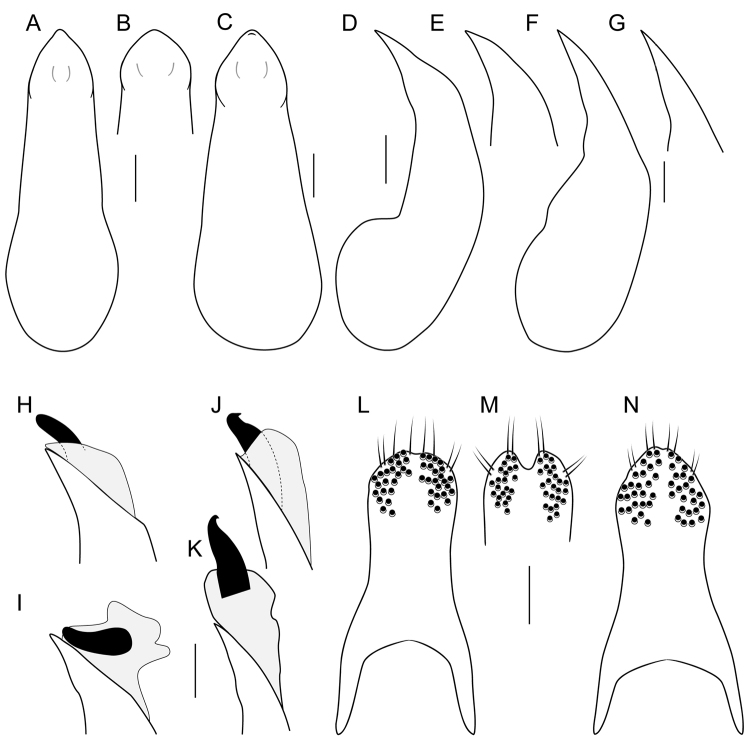
**A–K** median lobe of aedeagus **A–C** in ventral view **D–K** in lateral view **H, J** internal sac partly everted, showing paired ventral sclerites **I, K** completely everted **L–N** underside of paramere showing peg setae **A, B, D, E, H, I, L, M***Iratiquediusseriatus* (Horn) **C, F, G, J, K, N***I.uncifer* sp. nov. Scale bars: 0.1 mm.

**Figure 8.**
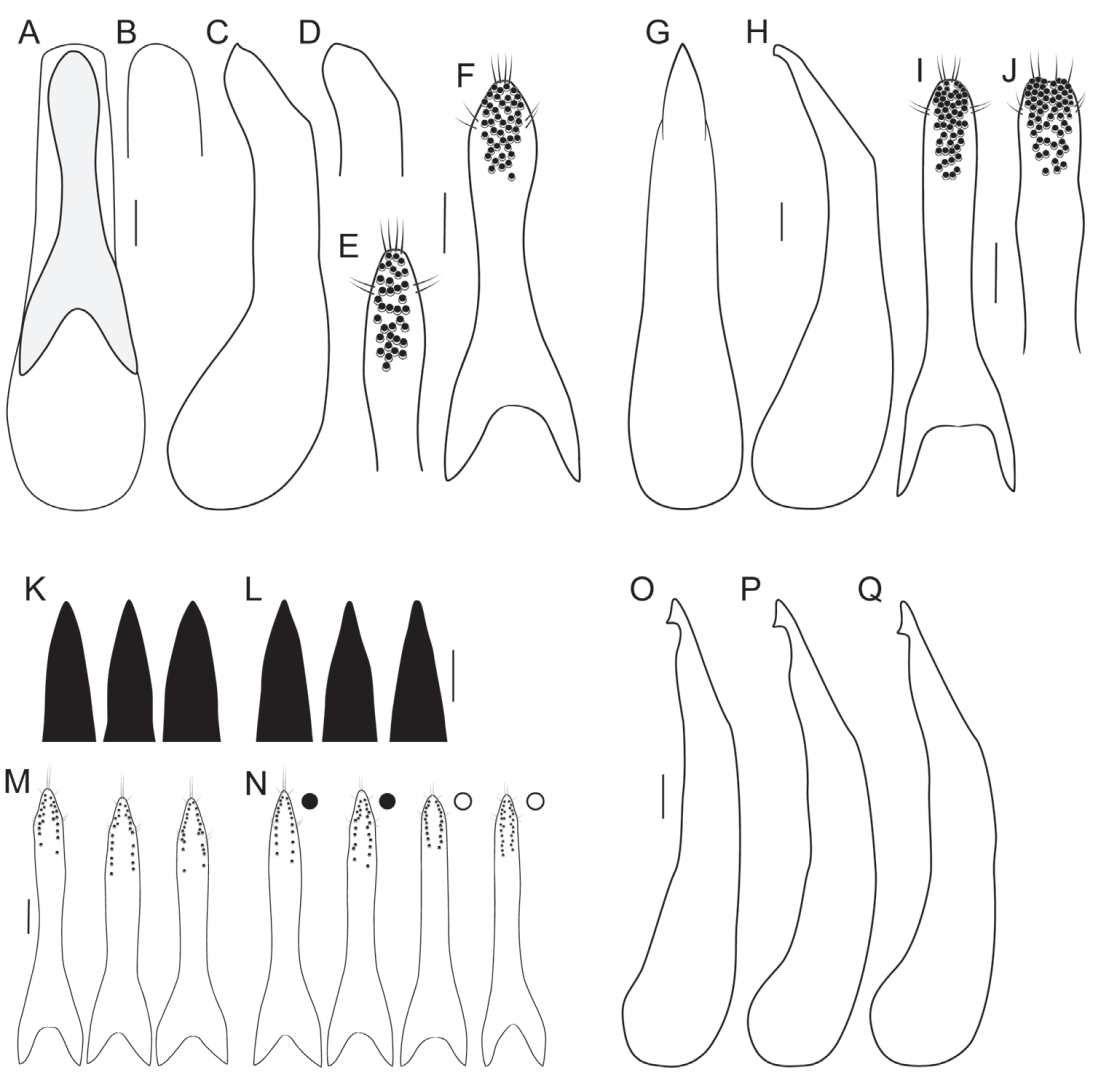
**A** aedeagus in ventral view **B, C, D, G, H, K, L** median lobe of aedeagus **B, G, K, L** in ventral view, **C, D, H, O–Q** in lateral view **E, F, I, J, M, N** underside of paramere showing peg setae **A–F***Paraquediusmarginicollis* sp. nov. **E** atypical specimen from Clallam County, Washington, United States **G–J***Paraquediuspuncticeps* (Horn) **I** lectotype, from Vancouver, British Columbia, Canada **J** non-type specimen from Vancouver Island **K–Q***Quediellusdebilis* (Horn), specimens from east (**K, M)** and west (**L, M)** sides of the continental divide. Filled circles = typical ‘nanulus’ morphotype; open circles = typical ‘debilis’ morphotype. Scale bars: 0.1 mm.

##### Distribution.

*Paraquedius* is endemic to western North America, from coastal British Columbia, along the Coast, Cascade and Sierra Nevada Ranges, and as far south as the San Bernardino Mountains of California (Smetana 1981).

##### Bionomics.

Most specimens were taken in water-soaked moss or algae-covered rocks at waterfalls, fast streams or freshwater outflows to ocean beaches. A few specimens were collected near a stream under stones on muddy ground covered in algae (Smetana 1971).

##### Comments.

The two available specimens from the San Bernardino Mountains were females and males should be sought to determine whether there are additional species present. Given that some specimens were collected at cold seeps amongst rather dry, scrubby forest, this genus may eventually be found further south in California, and potentially in the forested mountain valleys of Baja California and Baja California Sur, Mexico.

### ﻿Key to the species of *Paraquedius*

**Table:** 

1	Head with glabrous area extended posteriad to about middle of eyes (Fig. 5A); antennomere 1 entirely dark (Fig. 5A); pronotum and humeral area entirely dark (Fig. 2G); median lobe in lateral view strongly convergent to narrow apex (Fig. 8H); paramere elongate with apical part gradually narrowed (Fig. 8I, J); female tergite X with short, acute apex (Fig. 9F)	***P.puncticeps* (Horn)**
–	Head with glabrous area not extended posteriad to middle of eyes (Fig. 5B); antennomere 1 with pale base (Fig. 5B); margins of pronotum and at least extreme base of elytra paler, orange-red (Fig. 2H); median lobe in lateral view broad, narrowed only at the very apex (Fig. 8C, D); paramere spoon-shaped (Fig. 8E, F); female tergite X with truncate apex (Fig. 9G)	***P.marginicollis* sp. nov.**

#### ﻿ Paraquedius puncticeps

Taxon classificationAnimaliaColeopteraStaphylinidae

Horn, 1878, comb. res.

8533B342-2EDE-5979-92A9-8BC381EAE009

**Figure 9.**
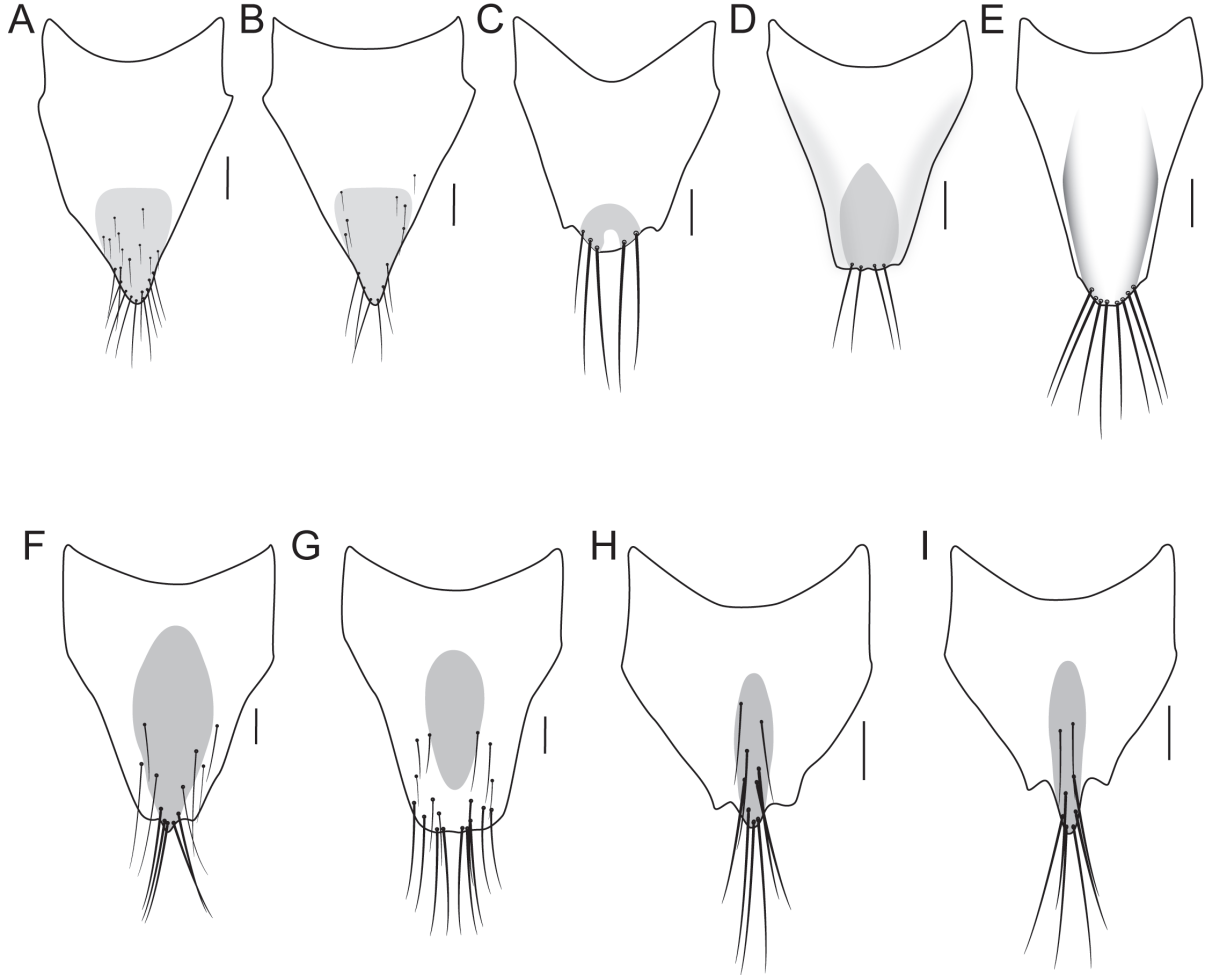
**A–I** female tergite X **A***Iratiquediusamabilis* (Smetana) **B***I.mutator* (Smetana) **C***I.prostans* (Horn) **D***I.seriatus* (Horn), evenly convex **E***I.uncifer* sp. nov., discal impression **F***Paraquediuspuncticeps* (Horn) **G***P.marginicollis* sp. nov. **H–I***Quediellusdebilis* (Horn). Pigmented area on tergite in **E** not shown for clarity but similar to *I.seriatus.* Scale bars: 0.1 mm.

Figs 2G , 5A , 8G–J , 9F , 11D (map)

Quedius puncticeps Horn, 1878: 156, 166.
Paraquedius puncticeps ; Casey 1915 (redescription).
Quedius (Paraquedius) puncticeps ; Smetana 1971 (redescription, partial misidentification of P.marginicollis).

##### Type locality.

Vancouver, British Columbia, Canada.

##### Type material.

***Lectotype* (male, MCZ – Horn coll.)**: ‘Vanc.’ [= Vancouver] [printed label] / LectoTYPE 3048 [red printed label] / Q. puncticeps H. [handwritten label] / MCZ00732196 [identifier] / LECTOTYPE *Quediuspuncticeps* det. A. Brunke 2022 [red printed label]

***Paralectotype* (male, MCZ – LeConte coll.)**: ‘Van.’ [printed label] / [male symbol, printed] / Type 7273 [red printed label] / Q. puncticeps H. [handwritten label] / PARALECTOTYPE *Quediuspuncticeps* det. A. Brunke 2022 [yellow printed label].

***Paralectotype* (female, MCZ – LeConte coll.)**: ‘Van.’ [= Vancouver] [printed label] / MCZ00732195 [identifier] / PARALECTOTYPE *Quediuspuncticeps* det. A. Brunke 2022 [yellow printed label].

##### Non-type material.

**Canada: British Columbia**: Vancouver Island: Goldstream Park, 5 mi N Victoria, 27.V.1968, Campbell and Smetana (2 females, CNC); Goldstream Park, 27.V.1968, A. Smetana (1 female, CNC); near Mount Finlayson Trail, Malahat, Goldstream Provincial Park, moss on rock, 11.VII.1979, I.M. Smith (1 male, 2 females, CNC); ~2.2 km W Shirley, on beach, algae-covered sandstone in freshwater outflow, 18.X.2018, iNaturalist observation 18374642 by user thomasbarbin. **United States: Washington**: ‘W.T.’ [= Washington Territory], 1 male (MCZ).

##### Diagnosis.

*Paraquediuspuncticeps* is most easily distinguished from the only other species of the genus by the entirely dark first antennomere and pronotum. For other differences see the key above.

##### Redescription.

Measurements ♂ (*n* = 4): HW/HL 1.06–1.07; PW/PL 1.01–1.04; EW/EL 1.09–1.18; ESut/PL 0.88–0.91; PW/HW 1.03–1.07; forebody length 4.2–4.5 mm.

Measurements ♀ (*n* = 5): HW/HL 1.05–1.13; PW/PL 1.05–1.08; EW/EL 1.12–1.21; ESut/PL 0.88–0.94; PW/HW 1.00–1.06; forebody length 4.3–4.9 mm.

Head, pronotum and elytra dark brown, slight metallic bronze-green reflection (Fig. 2G); antennae dark brown, antennomere 1 entirely dark, base of antennomere 2 paler, red to dark red (Fig. 5A); palpi dark brown; legs dark brown with paler, brownish yellow femora, tarsi often paler than tibiae; abdomen dark brown with narrowly to broadly paler apices.

Head slightly transverse, temples short and rather strongly rounded to neck, eyes bulging and distinctly protruding from lateral head margin; frons with central glabrous area extended posteriad to at least middle of eyes (Fig. 5A); disc of head with microsculpture of sparse, superficial transverse waves, often changing direction and forming irregular meshes especially on frons; head with moderately deep, circular impressions mediad of anterior frontal punctures (Fig. 5A); labrum short, transverse, forming two lobes; antennomeres 1–9 elongate, becoming shorter toward apex of antennae, antennomere 10 subquadrate; pronotum slightly transverse, roughly shield-shaped, varying from flattened with apex broader than neck, to distinctly convex with apex about as wide as neck; pronotum with microsculpture as on head, becoming meshed in places, especially on anterior angles; elytra moderately to distinctly transverse, appearing elongate, distinctly dilated at apex; disc without microsculpture, with sparse, moderately fine and even punctation, most punctures separated by 1.5–2.0 times the width of a puncture, disc weakly convex and uneven, most specimens with only weak discal impressions; with fully developed hind wings; abdominal tergites III–V with basal impression, tergites III–VI with paired, sparse lateral whorls of pale setae (visibility depends on lighting or condition of specimen); tergites with moderately sparse microsculpture of transverse waves, lines separated by at least their widths, tergites with very sparse punctation, punctures separated by many times their diameter.

Male with sternite VIII with moderately deep emargination, slightly less than twice as wide as deep; tergite X triangular, with narrow but broadly rounded apex; sternite IX rather stout, with asymmetrical base, rounded sides and narrow, non-emarginate apex; median lobe of aedeagus in ventral view strongly converging to acute apex, with slightly delimited subapical part (Fig. 8G); median lobe in lateral view strongly converging to elongate and curved apical part, apex narrow and slightly bent ventrad (Fig. 8H); paramere slightly longer than median lobe, elongate and sub-parallel, with slight middle and subapical expansions or evenly, gradually expanded to subapex, apex slightly emarginate and broad to entire and rounded, with numerous peg setae arranged into long median field that is narrowed basad (Fig. 8I, J). Female with tergite X elongate triangular, with short acute apex (Fig. 9F).

##### Distribution.

**Canada**: BC. **United States**: WA.

Thus far, *P.puncticeps* is known only from the Vancouver area and Vancouver Island, British Columbia, Canada, and from one male collected somewhere in Washington. More collecting in its preferred microhabitats is needed to determine the full distribution of *P.puncticeps*.

##### Bionomics.

Specimens have been collected in moss on rocks near a stream, under stones on muddy ground covered in algae (Smetana 1971) and on algae-covered sandstone in a freshwater outflow to the ocean (iNaturalist observation: https://www.inaturalist.org/observations/18374642).

##### Comments.

**Figure 10.**
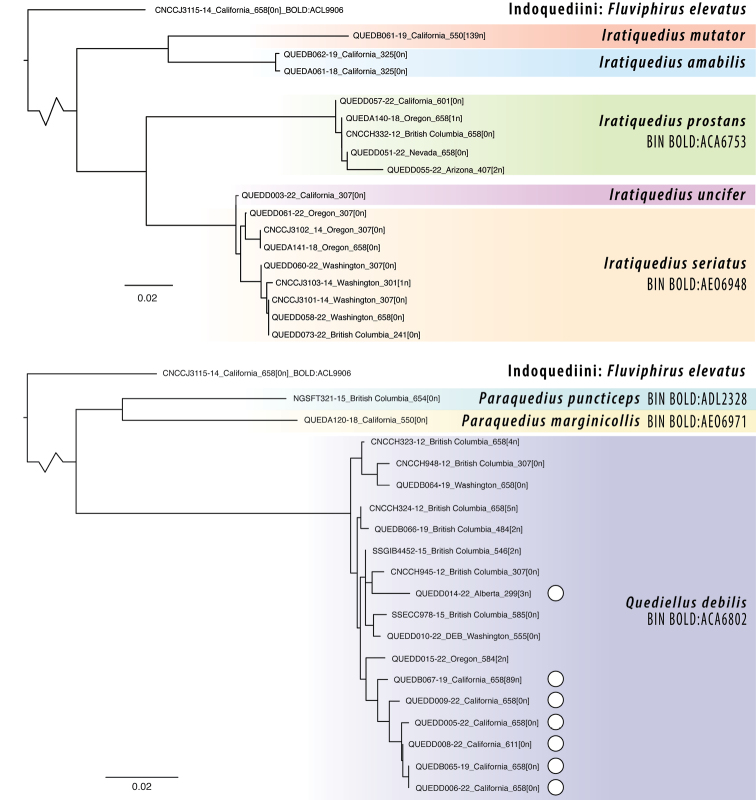
Neighbor-joining trees of CO1 barcode sequences for species of *Iratiquedius, Paraquedius*, and *Quediellus*, calculated under the Kimura-2 model for pairwise distance. White circles indicate macropterous ‘debilis’ morphotypes of *Q.debilis*, all other *Q.debilis* are of brachypterous ‘nanulus’ morphotypes. Scale bar equivalent to 2% divergence. Sequence length, including number of ambiguous base pairs (N’s), is given at the end of each sample name.

It is unusual in Staphylinidae for sister species to be sympatric but the morphological differences between the two known species of *Paraquedius* are so great that they may have diverged allopatrically long ago, with populations coming into secondary contact since then. Indeed, their CO1 barcodes are 8.79% different (Fig. 10).

#### ﻿ Paraquedius marginicollis sp. nov.

Taxon classificationAnimaliaColeopteraStaphylinidae

1B5D457B-4DC9-58EF-8B14-9A36C97702E8

https://zoobank.org/75E79E2A-CDE5-4FA2-A6F6-9BD36A2CAE1B

**Figure 11.**
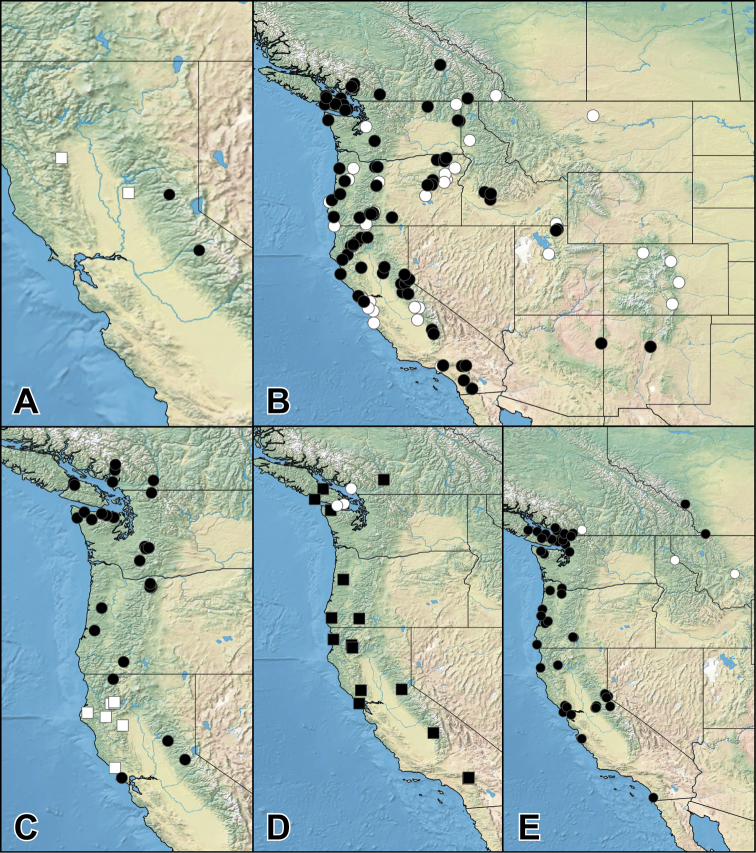
Distributions of **A***Iratiquediusamabilis* (Smetana) (circles), *I.mutator* (Smetana) (squares) **B***I.prostans* (Horn) (black – specimens examined, white – literature records) **C***I.seriatus* (Horn) (circles), *I.uncifer* sp. nov. (squares) **D***Paraquediuspuncticeps* (Horn) (circles), *P.marginicollis* sp. nov. (squares) **E***Quediellusdebilis* (Horn) (circles) (black – specimens examined, white – literature records).

Figs 2H , 5B , 8A–F , 9G , 11D (map)

Quedius (Paraquedius) puncticeps Horn (misidentification): Smetana 1981 (distribution records); Brunke et al. 2016, 2019, 2021 (phylogeny).

##### Type locality.

5.4 miles southeast of Seiad Valley [probably at O’Neil Creek], Siskiyou County, California, United States.

##### Type material.

***Holotype* (male, CNC)**: Siskiyou County, 5.4 mi SE Seiad Valley, 457 m, 4.VII.1976, #1272, L. & N. Herman [printed label] / CNC977001 [identifier] / HOLOTYPE *Paraquediusmarginicollis* Brunke, des A. Brunke 2022 [red printed label]. ***Paratypes* (19, CNC, FMNH, UTCI, MCZ).** Same data as holotype (2 females, CNC).

**Canada: British Columbia**: Vancouver Island: ‘V.I’ [no data] (1 female, CNC); Darling River, Pacific Rim National Park, 13.VII.1975, J.M. Campbell & B.A. Campbell (1 male, CNC); 10 mi E [Port] Alberni, MacMillan Provincial Park, 26.V.1968, Campbell and Smetana (1 female, CNC); Lower Mainland: Hells Gate, rest stop approx. 2 km S on Hwy 1, mountain stream, under weeds, 17.IX.1982, B & J. Carr (1 male, CNC).

**United States: California**: ‘Cal.’ (1 female, MCZ); El Dorado Co.: 0.7 mi E Pacific House, 38.758, -120.493, 1190 m, ex. screening flume, 18.VI.1989, A.R. Hardy & D. Carlson (1 male, UTCI); Humboldt Co.: Prairie Creek Redwoods State Park, ca. 2 mi S Fern Canyon, base of Gold Bluffs, 41.3748, -124.07, 10 m, scrubby coastal forest, on algae and vertical rock face at seep, 18.VI.2006, A. Newton & M. Thayer (1 female, FMNH); Marin Co.: Samuel P. Taylor State Park, 4.V.1968, Campbell and Smetana (1, CNC); Napa Co.: 10.1 mi N Calistoga, 579 m, flood debris, forest stream, 21.V.1976, A. Newton & M. Thayer (1 male, CNC); San Bernardino Co.: 1 mi SW [W] Mountain Home Village, 15.III.1983, A. Smetana (1 female, CNC); Camp Angelus [= Angelus Oaks], 1.VII.1970, K. Stephan (1 female, CNC); Trinity Co.: Upper Canyon Creek Meadows, 16 mi N Junction City, 1463 m, 13–19.VII.1979, J.M. & B.A. Campbell (1 female, CNC); Tulare Co.: Ash Mountain Power Station [Sequoia National Park], 23.XI.1982, J.A. Halstead (1, CNC); **Oregon**: Benton Co.: Mary’s Peak, 1158 m, 27.VII.1979, J.M. & B.A. Campbell (1 female, CNC); Curry Co.: Agness Rd., crossing at Wake Up Rilea Creek, under stones and in little pools of water along shady, cascading creek, 10.VIII.1978, B.F. & J.L. Carr (1 male, CNC); Jackson Co.: Highway 140, Little Butte Creek, 23.VI.1974, A. & D. Smetana (1 male, CNC); **Washington**: Clallam Co.: 6.5 miles N Sappho, 366 m, 16.VII.1978, #1669, L. & N. Herman (1 male, CNC.

All paratypes with: PARATYPE *Paraquediusmarginicollis* Brunke, des A. Brunke 2022 [yellow printed label]

##### Etymology.

The species epithet refers to the diagnostic pale margin of the pronotum.

##### Diagnosis.

*Paraquediusmarginicollis* is most easily distinguished from the only other species of the genus by the pale base of antennomere 1 and margins of the pronotum. For other differences see the key above.

##### Description.

Measurements ♂ (*n* = 5): HW/HL 1.04–1.09; PW/PL 1.01–1.08; EW/EL 1.13–1.17; ESut/PL 0.83–0.88; PW/HW 0.99–1.07; forebody length 4.0–4.3 mm.

Measurements ♀ (*n* = 5): HW/HL 1.06–1.10; PW/PL 1.01–1.08; EW/EL 1.11–1.17; ESut/PL 0.86–0.89; PW/HW 1.01–1.08; forebody length 4.3–4.5 mm.

Similar to *P.puncticeps* and differing only in the following: antennomeres 1 and 2 with pale base (Fig. 5B); marginal area of pronotum and at least extreme base of elytra, suture and sometimes scutellar area, paler (Fig. 2H); maxillary palpi usually slightly paler; head and pronotum with microsculpture more distinct; frons with central glabrous area not reaching posteriad to middle of eyes (Fig. 5B); frons with pair of impressions shallower and usually more linear, forming a border around raised central area (Fig. 5B); elytra with punctures slightly finer, disc always strongly uneven, with metallic greenish blue reflection; abdominal tergites with central, sparsely punctate to impunctate, raised areas, in addition to usual basal impressions; whorls of pale setae on tergites appearing more distinct from surrounding setae; male with emargination of sternite VIII slightly shallower, about twice as wide as deep; tergite X more slender, with narrower apex and setae more restricted to apical area; sternite IX with apex broadly, shallowly emarginate; median lobe of aedeagus in ventral view subparallel-sided, with truncate or broadly rounded apex (Fig. 8A, B); median lobe in lateral view with short apical area that is acute to obtusely pointed (Fig. 8C, D); paramere varying from slightly longer to slightly shorter than median lobe, spoon-shaped to lancet-shaped (Fig. 8E, F), peg setae forming long, oval-shaped median field (Fig. 8E, F). Female with tergite X shorter, with apex broader and truncate (Fig. 9G).

##### Distribution.

**Canada**: BC. **United States**: CA, OR, WA.

##### Bionomics.

Specimens with microhabitat data were collected at a variety of elevations (near sea-level to 1463 m), on or under surfaces (under rocks, on vertical rockface), in association with running water, including mountain streams, vertical seeps and waterfalls. The specimen from flood debris was likely washed out of its normal microhabitat by heavy rains.

##### Comments.

Initially, it was thought that the specimen from Clallam County, Washington represented yet another species, as the paramere is remarkably lancet-like (Fig. 8E) and longer than the median lobe, while most specimens have a spoon-shaped paramere that is shorter than the median lobe. With further dissections, a few intermediate forms were found, though this specimen still represents the extreme of variation.

#### ﻿ Quediellus

Taxon classificationAnimaliaColeopteraStaphylinidae

Casey, 1915, stat. res.

727C0873-0C0A-55D5-8AA2-7AE059DAE8B2

Figs 5C, D , 8K–Q , 9H, I

Quediellus Casey, 1915: 398, 402; Smetana 1971 (as synonym of Quedius (Raphirus)), Brunke et al. 2021 (phylogeny, isolated position, non-Raphirus, to be reinstated as genus).

##### Type species.

*Quediusdebilis* Horn, 1878.

##### Diagnosis.

*Quediellus*, in the restricted sense used here, can be recognized within Quediini by a combination of: head with genal and interocular punctures absent; pronotum without extra punctures between dorsal and sublateral rows, sublateral rows not extended posteriad of single large lateral puncture (but sometimes at same level); prosternum without trace of longitudinal carina; scutellum impunctate; elytra with punctures not arranged in distinct rows, spaces between with distinct meshed microsculpture (Fig. 5D). *Quediellus* and *Quedionuchus* are the only genera of Quediini with meshed (scale-like) microsculpture on the elytra (Fig. 5D, E), while other lineages may have granulose microsculpture composed of micropunctures or microsetae (Fig. 5F), superficially giving a similar dull appearance at low magnification. *Quediellus* differs from *Quedionuchus* by the irregularly scattered (not rows) or evenly distributed elytral punctures (Fig. 5D versus Fig. 5E) and complete infraorbital ridge, running from the neck to the base of the mandibles under the eye.

*Quediellus* shares plesiomorphic, simple head chaetotaxy (though the basal puncture is often doubled, e.g., Fig. 5C) with members of the mostly Palaearctic subgenus Quedius (Raphirus) (sensu Brunke et al. 2021), with which it was long considered to be synonymous. However, all Quedius (Raphirus) differ by the lack of meshed microsculpture on the elytra and those with a dull reflection between the punctures (e.g., *Q.cincticollis* Kraatz, *Q.fumatus* (Stephens), the members of clade ‘X2’ of Brunke et al. 2021 (*Q.lateralis* (Gravenhorst) and its relatives)) have micropunctures rather than meshes, much denser elytral punctation and most have an, at least partly, carinate prosternum.

##### Redescription.

**Figure 12.**
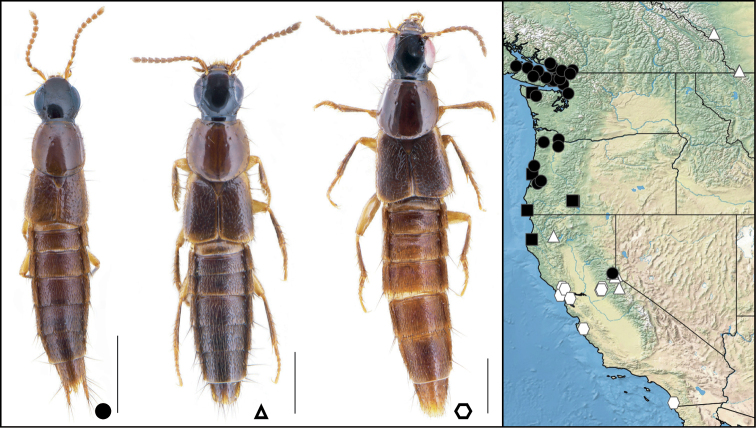
Habitus and distribution of *Quediellusdebilis* (Horn) morphotypes: brachypterous, palisade fringe absent (circle), brachypterous, palisade fringe present (square – habitus as in previous), winged, shorter and narrower elytra (triangle), winged, longer and broader elytra (hexagon). Scale bar = 1 mm.

Small and slender, to medium-sized and fusiform rove beetles, often with pale yellow markings on apex, humerus and sides of elytra (Fig. 12). With the character states of Quediini (see Brunke et al. 2021) and the following: antennomere 3 longer than 2, without dense setae; head with eyes large, much more than twice as long as temples, convex, bulging from lateral head outline, weakly convergent anteriad and with inner margin well separated from suprantennal ridge (Fig. 5C); antennal insertions close to inner margin of eye, separated by about the width of antennal sclerite (Fig. 5C); frons weakly developed anterolaterad of antennal insertions; with single or doubled basal puncture; interocular, parocular and genal punctures absent; labrum notched medially, creating two short lobes; apical maxillary and labial palpi fusiform and glabrous; infraorbital ridge complete to mandibles; gular sutures converging towards neck and narrowly spaced posteriad; mandibles with dorsal lateral groove; right mandible with single, simple tooth (Fig. 5C); pronotum subquadrate; hypomeron strongly inflexed, not visible in lateral view; with single large lateral puncture; dorsal row of pronotum with three punctures; sublateral row not reaching level of large lateral puncture; basisternum with pair of distinct macrosetae, without trace of longitudinal carina; elytron with subbasal ridge complete, forming scutellar collar; scutellum impunctate; with row of three or four humeral spines; disc of elytra with even punctation, with distinct meshed microsculpture; foretibia with lateral spines and apical spurs; metatarsomeres with disc setose; metatibia spinose with at least three spines on outer face; abdominal tergite I with prototergal glands well developed, with one side surrounded by row of well-developed setae; abdominal tergites without impressed, glabrous basal areas; abdominal sternite III with basal transverse carina forming obtuse angle at middle, not produced; abdominal sternite VII unmodified; abdominal sternite VIII with distinct median emargination; aedeagus with well-developed paramere bearing peg setae (Fig. 8M, N).

##### Distribution.

*Quediellus* is endemic to the western Nearctic, occurring along the western cordilleras from southern British Columbia to southern California on the western side of the continental divide, and known from the Rocky Mountains of Alberta, Idaho and Montana on the eastern side.

##### Bionomics.

Specimens have been collected mainly from sifting leaf litter, rotting wood and moss along streams, in forests and in montane meadow.

##### Comments.

Casey (1915) erected the genus *Quediellus* to unite species belonging to the Debilis and Brunnipennis groups of Smetana (1971), based on an entire labrum. This concept was correctly recognized by Smetana (1971) as erroneous as not only did he consider these two groups to be distantly related but only *Quediusdensiventris* exhibited an entire labrum and only in some individuals. The always bilobed labrum of *Quediellus* is quite transverse in some specimens and sometimes at lower magnification it can be difficult to observe its shape. The type species of *Quediellus*, *Q.debilis*, was assigned to the Debilis group of Quedius (Raphirus) by Smetana (1971) and has been treated as such ever since. Quite recently (Brunke et al. 2021), the Debilis group (as *Quediusnanulus* (Casey)) was shown to be one of the smaller, phylogenetically isolated lineages of Quediini and quite distantly related to true Quedius (Raphirus), despite sharing several morphological, though plesiomorphic, character states including the simple head chaetotaxy. In order to achieve monophyly of both *Quedius* and subgenus Raphirus, *Quediellus* is here resurrected as a valid genus under a morphological concept that is similar to that given by Smetana (1971) for the Debilis group.

#### ﻿ Quediellus debilis

Taxon classificationAnimaliaColeopteraStaphylinidae

(Horn, 1878), comb. res.

E8888305-7D03-55F9-A33B-62C5A65F7029

Figs 5C, D , 8K–Q , 9H, I , 11E (map), 12

Quedius debilis Horn, 1878: 156, 165.
Quediellus debilis ; Casey 1915 (redescription).
Quediellus helenae Casey, 1915: 403 syn. nov.
Quediellus humilis Casey, 1915: 403 syn. nov.
Quediellus nanulus Casey, 1915: 402 syn. nov.
Quedius (Raphirus) debilis ; Smetana 1971 (redescription); Brunke et al. 2021 (phylogeny, as Q.nanulus).

##### Type locality.

California (unknown locality), United States.

##### Type material.

***Lectotype* (female, MCZ, examined digitally)**: ‘Cala’ [white typed label] / Q.debilis H. [handwritten label] / Type 7270 [red label].

The female lectotype of this species is a classic example of the ‘debilis’ morphotype (see below): a larger specimen with elytra at sides much longer than pronotum at midline, elytra at apex wider than pronotum.

#### ﻿ Quediellus nanulus

Taxon classificationAnimaliaColeopteraStaphylinidae

Casey

D2895A91-D95D-5E56-9174-1D772A742706

##### Type material.

***Lectotype* (male, United States National Museum of Natural History, not examined)**: Lane Co. Or / Casey bequest 1925 / Type USNM 48307 / nanulus Csy.

Smetana (1971) stated that the male lectotype was a typical member of *Q.nanulus* (e.g., short, narrow elytra, small-bodied). Several non-type male specimens from the type locality matching this description and consistent with the ‘nanulus’ morphotype, were available for study. The aedeagi of these specimens fell within the range of morphological variation considered to belong to a single, variable *Q.debilis*.

#### ﻿ Quediellus helenae

Taxon classificationAnimaliaColeopteraStaphylinidae

Casey

423F50EA-AF31-55F5-8AF3-447C3EE39182

##### Type material.

***Holotype* (female, United States National Museum of Natural History, not examined)**: “Helena Mont.” / Casey bequest 1925 / Type USNM 48305 / helenae Csy.

Smetana (1971) stated that the male lectotype was also a typical member of *Q.nanulus*. All available specimens from the Rockies were small-bodied, winged, with palisade fringe and narrow elytra. Subtle differences in the aedeagus were observed between individuals on either side of the continental divide (see Redescription below) but the single available barcode sequence from the east clustered deep within the western sequences of *Q.debilis*.

#### ﻿ Quediellus humilis

Taxon classificationAnimaliaColeopteraStaphylinidae

Casey

C806BB11-7C43-5FA9-8122-B5BFAF3C1ED3

##### Type material.

***Holotype* (male, United States National Museum of Natural History, not examined)**: “Cal”. / Casey bequest 1925 / Type USNM 48306 / humilis Csy. Smetana (1971) mentioned that this specimen was a small male of *Q.nanulus*. While it was not studied, several small non-type males were examined from northern California that perfectly fit the ‘nanulus’ morphotype, including some examined by Smetana (1971) himself and identified as *Q.nanulus*.

##### Non-type material.

**Canada: Alberta**: Marmot Creek, 10 mi SW Kananaskis F.E.S., 15.VIII.1971, 5000’, sifting deciduous litter along large stream, J.M. Campbell (1 male, 1 female, CNC); Waterton Lakes N.P., mi 2 of Red Rock Canyon Road, 16.VI.1980, 4400’, J.M. Campbell (1 male, 1 female CNC); Waterton Lakes N.P., 1 mi N Pass Creek Bridge, 4500’, sifting *Populus* litter in moist deciduous forest, 5.VIII.1976, J.M. Campbell (1 male, 1 female, CNC). **British Columbia**: Greater Vancouver: Stanley Park, 20.V.1989, A. Smetana (2, CNC); same except 28.V.1968, Campbell and Smetana (1, CNC); same except 21.V.1968, Campbell and Smetana (1, CNC); Brunswick, 20.V.1968, Campbell and Smetana (1, CNC); Langley, open oak forest, 10.XII.1979, S. Rahe (2, CNC); Fraser Valley: Mission, berlese sample, 25.VII.1953, W.R.M. Mason (2, CNC); Vancouver Island: Chemainus Lake, mushrooms and rotten fir wood, edge of path, 25.IX.2020, A. Davies (1, CNC); Mesachie Lake, Forest Experiment Station, 1950 m, woody debris from log, 23.VII.1979, I. Smith (2, CNC); Salt Spring Island, Fulford Harbour, Petroglyph, 25.IX.1974, BD Ainscough (2, CNC); Gabriola Island, Gabriola, marsh with sedges, pitfall traps, 30.IX.1994, BF & JL Carr (1, CNC); same except sifting maple litter beside firewood and compost bin, 30.IV.1993 (3, CNC); same except sifting moss in forest near Hoggan Lake, 14.VI.1989 (1, CNC); Englishman River Provincial Park, cedar litter, 10.III.1997, B.D. Ainscough (1, CNC); Yellow Point, Cedar, under rotten log, 18.V.1978, B.D. Ainscough (1, CNC); Lake Cowichan, spring run beside North Shore Road, 1.7 km N town, moss and litter, 7.VI.1979, I. Smith (1, CNC); Stamp Falls [Stamp River] Provincial Park, 8 mi NW Alberni, 26.V.1968, Campbell and Smetana (2, CNC); Victoria, sifting litter and grass, 28.X.1985, BF & JL Carr (1, CNC).

**United States: California**: Alpine Co.: 22 mi NE Strawberry, Clark Fork River near Cottonwood Creek, 1767 m, 14.VII.1976, L. & N. Herman (1, CNC); Amador Co.: 4 mi S Jackson, sift oak, maple and alder litter, 3.II.1979, D.S. Chandler (1, FMNH); 1 mi W Pine Grove, 24.VI.1975, A. Newton and M. Thayer (1, CNC); ‘Mokel Hill’ [= Mokelumne Hill], VII.1925, VanDyke (4, CNC); Alameda Co.: Berkeley, under Umbellifera duff, 23.II.1962, J.F. Lawrence (1, CNC); El Dorado Co.: Lake Tahoe, Taylor Creek at Camp Richardson, 1900 m, 9.VII.1986, A. Smetana (2, CNC); Humboldt Co.: 5 mi N Trinidad, Patricks Point State Park, hemlock/pine/alder litter, 14.VIII.1966, J+S Cornell (1, FMNH); Eureka, VI.1902, H.S. Barber (2, CNC); Marin Co.: Lagunitas Creek, near Tocaloma, 18.III.1983, A. Smetana (5, CNC); Monterey Co.: Jamesburg, Ian Moore (1, CNC); Placer Co.: Meeks Bay, West Lake Blvd, 1917 m, ex. riparian litter, 15.XI.2008, A. Brunke (1, DEBU); Lake Tahoe, Tahoe City, 1950 m, 7.VII.1986, A. Smetana (2, CNC); Tahoe Pines, 3.V.1968, Campbell and Smetana (1, CNC); Tahoe Pines, 1889 m, 10.VIII.1969, A. Smetana (2, CNC); San Diego Co.: San Diego, F.E. Blaisdell (1, CNC); Sonoma Co.: Annadel State Park, Hunter Spring nr. Canyon Trail, 220 m, 26.IV.–17.V.2007, P. Kerr and S. Blank (1, UTCI); Sobre Vista, 24.IV.1910, Van Dyke (1, CNC); Trinity Co.: Upper Canyon Creek Lake, 17mi N Junction City, 1737 m, 12.VII.1979, JM & BA Campbell (6, CNC); Upper Canyon Creek Meadows, 16mi N Junction City, 1463 m, 13–19.VII.1979 (1, CNC); **Nevada**: Douglas Co.: Zephyr Cove, 1900 m, 9.VII.1986, A. Smetana (3, CNC); **Oregon**: Columbia Co.: Oak Island, Sauvies Lake, Columbia River, 5mi N 2mi E Burlington, 30 m, Oregon oak and snowberry duff, 7.X.1972, E.M. Benedict (2, CNC); Curry Co.: 4mi S Pistol River, on US 101 (Mark 342), 60 m, Sitka spruce duff and soil, 12.II.1972, E.M. Benedict (1, CNC); Douglas Co.: 1mi S & 2mi W Ash, 152 m, deciduous Myrtle litter and duff, 11.XII.1971, E.M. Benedict (1, CNC); ~4.5mi E Wells Creek, Ranger Station, Umpqua River, 91 m, oak duff and soil, 11.XII.1971, E.M. Benedict (1, CNC); Klamath Co.: Mares Egg Springs, 1280 m, 20.VII.1979, JM Campbell & J Schuh (4, CNC); same except 25.VI.1974, A. & D. Smetana (1, CNC); 7 mile Creek, west of Forth Klamath, 20.VII.1979, JM Campbell & J Schuh (1, CNC); Lane Co.: Coast Guard Road, Approximately 1mi N Florence, 152 m, moss festoons, 6.V.1972, E.M. Benedict (2, CNC); Lincoln Co.: Eakman [= Ekman] Lake, 5mi E Waldport, 0 m, beach grass, 5.V.1973, E.M. Benedict (2, CNC); Tillamook Co.: Rest area, Wilson River Highway, 0.5mi S & 1mi W Lee’s Camp, western red cedar and red alder, 4.XI.1972, E.M. Benedict (2, CNC); Washington Co.: 0.25mi E Sherwood Highway & US-99W, 60 m, rotten hay, barley and dung, 1.I.1972, E.M. Benedict (2, CNC); **Washington**: Jefferson Co.: Olympic National Park, Hoh Ranger Station, 182 m, 13.V.1968, Campbell and Smetana (1, CNC); same except 19.VIII.1979, JM & BA Campbell (4, CNC); San Juan: San Juan Islands, Jones Island, 2.V.1988, J. Bergdahl (2, CNC); San Juan Islands, Matia Island, 28.VIII.1988, J. Bergdahl (2, CNC); San Juan Islands, Flattop Island, 22.I.1988, J. Bergdahl (1, CNC); San Juan Islands, Stewart Island, 18.X.1987, J. Bergdahl (1, CNC); same except 6.VIII.1988 (1, CNC); Sucia Island, 28.VIII.1988, J. Bergdahl (4, CNC); same except 30.VIII.1988; San Juan Islands, Jones Island, 2.V.1988, J. Bergdahl (3, CNC); San Juan Islands, Clark Island, 27.VIII.1988, J. Bergdahl (1, CNC); Skagit Co.: San Juan Islands, Fidalgo Island, 5.IV.1986, J. Bergdahl (4, CNC); Snohomish Co.: Chase Lake, 15.VIII.1961, W. Suter (2, CNC); Siskiyou Co.: VII, C.V. Riley (2, CNC); Whatcom Co.: Deming, S end Sumas Mt., Reardon property, ANMT site 1275, 95 m, berl., leaf & log litter incl. under *Phlebia* polypores on log, 2^nd^ growth *Pseudotsuga*-*Thuja*-*Alnus*-*Acermacrophyllum* & *circinatum*, 13.XI.2014, A. Newton & M. Thayer (1, FMNH).

##### Diagnosis.

As in generic diagnosis.

##### Redescription.

Measurements ♂ (*n* = 10 (5 macropterous; 5 brachypterous)): HW/HL 1.07–1.17; PW/PL 1.02–1.19; EW/EL 1.08–1.29; ESut/PL 0.54–0.79; PW/HW 1.14–1.27; forebody length 2.3–3.1 mm.

Measurements ♀ (*n* = 10 (5 macropterous; 5 brachypterous)): HW/HL 1.10–1.15; PW/PL 1.04–1.14; EW/EL 1.15–1.36; ESut/PL 0.56–0.82; PW/HW 1.14–1.30; forebody length 2.5–3.2 mm.

Head dark brown, darker than pronotum, which is paler, entirely brown to pale reddish with yellow borders; elytra brownish, often with epipleural area, and lateral and apicolateral parts of disc paler, frequently contrasting yellow; abdomen dark brown, tergites at most narrowly paler at apex; antennae brown, with antennomeres 1–3 paler, yellow-brown or at least bases paler; legs pale, yellow to yellowish brown, tibiae dark brown; palpi yellowish brown to dark brown (Fig. 12).

Head slightly transverse, eyes large, moderately convex and protruding from lateral outline, temples small, about 1/4 to 1/3 of eye length (Fig. 5C); disc of head with sparse microsculpture of transverse waves, spaces between lines greater than width of lines; antennomeres of varying shape depending on morphotype, with ‘nanulus’ type having 1–3 elongate, 4 quadrate to elongate, 5 or 6–10 transverse and macropterous individuals of the ‘debilis’ morphotype having 1–5 or 1–6 elongate and 7–10 or 8–10 distinctly transverse; pronotum about as wide as long to moderately transverse, moderately wider than head; disc with microsculpture as on head; elytra moderately to distinctly transverse, markedly variable from scarcely longer at sides than pronotum at middle to moderately longer (1.06–1.21, ‘nanulus’ morphotype), or distinctly longer than pronotum at middle (1.26–1.41, ‘debilis’ morphotype) (Fig. 12); disc of elytra with punctures varying from superficial, sparse and widely scattered (nanulus type) to slightly denser and coarser in individuals with the longest elytra (Fig. 12); wings as non-functional stubs or fully developed; abdomen generally sparsely punctate, denser at bases of tergites but variable, with punctation overall denser in ‘debilis’ morphotype; abdomen with dense, very fine microsculpture of transverse waves; abdominal tergite VII without or with palisade fringe.

**Male.** Sternite VIII with distinct emargination of variable depth and width; tergite X triangular, with moderately long, narrowly rounded apex; sternite IX varying from slightly expanded to broad at middle, with long, slender asymmetrical basal part and narrow, rounded apex; median lobe in ventral view with acute apex, sides of apex straight to acuminate resulting in a pinched appearance (Fig. 8L), individuals from the Rockies with sides broadly arcuate (Fig. 8K); median lobe in lateral view bearing a distinct tooth of variable distance from apex and with variably-sized emargination ventrad of tooth, apex narrow and rounded (Fig. 8O–Q); paramere slender and varying from fusiform (basal constriction, widest subapically) to subparallel-sided or weakly narrowing apicad (Fig. 8M, N); paramere with peg setae arranged in two rows, varying from well-organized to slightly disorganized and doubled in places, rows divergent from sides of paramere and weakly to strongly convergent basad in western individuals (Fig. 8N); in eastern individuals, rows generally following the margins of paramere and not convergent basad (Fig. 8M).

**Female.** Tergite X overall pentagonal, with emargination on each side of protruding, pointed apex, with longitudinal, median pigmented area, setae generally limited to midline (Fig. 9H–I).

##### Distribution.

**Canada**: AB, BC. **United States**: CA, ID, MT, NV, OR, WA.

Broadly distributed along the western cordilleras on both the eastern and western sides of the continental divide (Fig. 11E). At the northern edge of its western distribution, *Q.debilis* is entirely flightless and appears to extend eastward along the Fraser River in British Columbia to at least the Hope area but it is not clear how distantly east and west populations are separated by the drier, central interior, if at all.

##### Bionomics.

In the northern part of its range on the western side of the continental divide (British Columbia to Oregon, Fig. 12), *Q.debilis* is entirely flightless (wings as small stubs) and lives more or less in moist, forest litter-based microhabitats, including leaf litter, moss on rocks, treehole litter, and decaying wood and fungi, though it is sometimes collected in litter or moss along the water’s edge. Further south, south of northernmost California (Fig. 12), the species is more typically found in wet litter along creeks and most commonly fully winged with longer elytra. On the eastern side of the continental divide, examined specimens (all from Alberta) were fully winged with palisade fringe but with narrow elytra. In Alberta, specimens were collected in deciduous litter (*Populus* sp.) in a forest and along a stream.

##### Comments.

Smetana (1971) treated *Quediellusnanulus* (as *Quedius*) as a separate though variable species that could be confidently diagnosed from *Q.debilis* solely by the ‘smaller aedeagus’ and in the key by the shorter and less narrow paramere with peg setae rows shorter. A more northern population of generally narrower, smaller-bodied individuals with shorter elytra was considered to correspond to *Q.nanulus*, while a more southern population of generally larger, more fusiform specimens with longer and broader elytra was considered to correspond to *Q.debilis*. Based on the material Smetana (1971) had available, there was some degree of geographic overlap in California, especially the northern Sierra Nevada (Lake Tahoe area).

An examination of dissected males from across this range on the western side of the continental divide, including many of the original specimens examined by Smetana (1971), revealed no reliable differences in the highly variable aedeagus in ventral or lateral view (but see below), despite remarkably disparate morphological forms at either end of the range of variability (Fig. 12). On average, individuals in the north of the distribution tend to have a paramere with a distinct basal constriction and long apical part, while individuals in the far south of the distribution have a more parallel-sided paramere with a shorter apical part (Fig. 8N). However, there are exceptions, even within the same series. These trends do not correspond with the drawings presented in Smetana (1971) for *Q.nanulus* (northern) and *Q.debilis* (southern) and it is possible that they were reversed. The length of the rows of peg setae, number of peg setae and their distribution were all found to be highly variable and did not correspond to external morphotype. Often this variation was marked, even among individuals from the same locality, despite rather uniform external morphology. Congruently, sequenced individuals of the ‘debilis’ and ‘nanulus’ morphotypes (Fig. 10, white circles) did not form separate clusters.

Individuals on the eastern side of the continental divide (Rocky Mountains) were considered by Smetana (1971) to belong to *Q.nanulus*. The three specimens examined (all Alberta) exhibited very subtle differences on the median lobe and paramere compared to all other western specimens (Fig. 8), though these differences may not hold when more males are dissected. Cluster analysis treated all specimens of *Quediellus* as a single OTU and BIN, with relatively high (3.27%) intraspecific divergence, likely due to the fact that this species is flightless and a poor disperser over much of its range (British Columbia to northern California). The single half-length sequence from the Rockies did not cluster separately from the western samples (Fig. 10). Therefore, *Q.nanulus* is here treated as a synonym of *Q.debilis* and both populations on either side of the continental divide are considered to be conspecific, leaving only one highly variable species of *Quediellus*.

## Supplementary Material

XML Treatment for Quediini

XML Treatment for Iratiquedius

XML Treatment for Iratiquedius amabilis

XML Treatment for Iratiquedius mutator

XML Treatment for Iratiquedius prostans

XML Treatment for Iratiquedius seriatus

XML Treatment for Iratiquedius uncifer

XML Treatment for Paraquedius

XML Treatment for Paraquedius puncticeps

XML Treatment for Paraquedius marginicollis

XML Treatment for Quediellus

XML Treatment for Quediellus debilis

XML Treatment for Quediellus nanulus

XML Treatment for Quediellus helenae

XML Treatment for Quediellus humilis
